# A compendium of human gene functions derived from evolutionary modelling

**DOI:** 10.1038/s41586-025-08592-0

**Published:** 2025-02-26

**Authors:** Marc Feuermann, Huaiyu Mi, Pascale Gaudet, Anushya Muruganujan, Suzanna E. Lewis, Dustin Ebert, Tremayne Mushayahama, Gene Ontology Consortium, Marc Feuermann, Huaiyu Mi, Pascale Gaudet, Anushya Muruganujan, Dustin Ebert, Tremayne Mushayahama, Suzanne A. Aleksander, James Balhoff, Seth Carbon, J. Michael Cherry, Harold J. Drabkin, Nomi L. Harris, David P. Hill, Raymond Lee, Colin Logie, Sierra Moxon, Christopher J. Mungall, Paul W. Sternberg, Kimberly Van Auken, Paul D. Thomas, Paul D. Thomas

**Affiliations:** 1 Swiss-Prot Group, SIB Swiss Institute of Bioinformatics, Centre Medical Universitaire, Geneva, Switzerland; 2 Division of Bioinformatics, Department of Population and Public Health Sciences, University of Southern California Los Angeles, Los Angeles, CA USA; 3 Environmental Genomics and Systems Biology, Lawrence Berkeley National Laboratory, Berkeley, CA USA; 4 Department of Genetics, Stanford University, Stanford, CA USA; 5 Renaissance Computing Institute, University of North Carolina, Chapel Hill, NC USA; 6 The Jackson Laboratory for Mammalian Genomics, Bar Harbor, ME USA; 7 California Institute of Technology, Pasadena, CA USA; 8 Faculty of Science, Radboud Institute for Molecular Life Sciences, Nijmegen, the Netherlands; 9 Texas A&M University, College Station, TX USA; 10 Northwestern University, Chicago, IL USA; 11 University of Maryland School of Medicine, Baltimore, MD USA; 12 Department of Physiology, Development and Neuroscience, University of Cambridge, Cambridge, UK; 13 University College London, London, UK; 14 Department of Biochemistry, University of Cambridge, Cambridge, UK; 15 NYU Grossman School of Medicine, New York, NY USA; 16 Medical College of Wisconsin, Milwaukee, WI USA; 17 Department of Fundamental Neurosciences, University of Lausanne, Lausanne, Switzerland; 18 Center for Molecular Neurobiology, ZMNH, University MC Hamburg-Eppendorf, Hamburg, Germany; 19 Leibniz Institute for Neurobiology, Magdeburg, Germany; 20 Department of Biomedicine and Prevention, University of Rome Tor Vergata, Rome, Italy; 21 Institut d’Investigació Biomèdica Sant Pau (IIB SANT PAU), Barcelona, Spain; 22 Universitat Autònoma de Barcelona, Bellaterra, Spain; 23 Department of Neurology, Yale School of Medicine, New Haven, CT USA; 24 Department of Molecular Neurobiology, Max Planck Institute for Multidisciplinary Sciences, Göttingen, Germany; 25 Yong Loo Lin School of Medicine, National University of Singapore, Singapore, Singapore; 26 Institute of Molecular and Cell Biology, Agency for Science Technology and Research (A*STAR), Singapore, Singapore; 27 Zilkha Neurogenetic Institute, University of Southern California, Los Angeles, CA USA; 28 Center for Neurogenomics and Cognitive Research, Amsterdam University Medical Center, Amsterdam, The Netherlands; 29 Sorbonne Université, Institut du Cerveau, Paris, France; 30 Kavli Neuroscience Discovery Institute, Johns Hopkins University, Baltimore, MD USA; 31 Center for Behavioral Brain Sciences (CBBS) and Institute of Pharmacology and Toxicology, Medical Faculty, Otto von Guericke University, Magdeburg, Germany; 32 Department of Neuroscience, University of Copenhagen, Copenhagen, Denmark; 33 Laboratory of Neurobiology, Max-Planck Institute for Biophysical Chemistry, Göttingen, Germany; 34 Department of Biological Sciences, Korea Advanced Institute of Science and Technology (KAIST), Daejeon, South Korea; 35 Department of Neurobiology, Harvard Medical School, Boston, MA USA; 36 Center for Neurogenomics and Cognitive Research, Vrije Universiteit Amsterdam, Amsterdam, The Netherlands; 37 Department of Molecular Physiology and Cell Biology, Leibniz-Forschungsinstitut für Molekulare Pharmakologie, Berlin, Germany; 38 Cell Biology, Neurobiology and Biophysics, Faculty of Science, Utrecht University, Utrecht, The Netherlands; 39 Department of Neurology and Neurosurgery, Montreal Neurological Institute, McGill University, Montreal, Quebec Canada; 40 Biological Sciences, University of Southampton, Southampton, UK; 41 Institute for Pharmacology and Toxicology, Otto-von-Guericke University, Magdeburg, Germany; 42 Department of Biochemistry, Weill Cornell Medicine, New York, NY USA; 43 CNR Neuroscience Institute Milan, Vedano al Lambro, Italy; 44 Stanley Center for Psychiatric Research, Broad Institute of MIT and Harvard, Cambridge, MA USA; 45 Department of Brain and Cognitive Sciences, Massachusetts Institute of Technology, Cambridge, MA USA; 46 Phoenix Bioinformatics, Newark, CA USA; 47 European Molecular Biology Laboratory, European Bioinformatics Institute, Hinxton, UK; 48 Cincinnati Children’s Hospital Medical Center, Cincinnati, OH USA; 49 University of Oregon, Eugene, OR USA

**Keywords:** Gene ontology, Functional genomics, Molecular evolution, Phylogeny, Evolutionary biology

## Abstract

A comprehensive, computable representation of the functional repertoire of all macromolecules encoded within the human genome is a foundational resource for biology and biomedical research. The Gene Ontology Consortium has been working towards this goal by generating a structured body of information about gene functions, which now includes experimental findings reported in more than 175,000 publications for human genes and genes in experimentally tractable model organisms^1,2^. Here, we describe the results of a large, international effort to integrate all of these findings to create a representation of human gene functions that is as complete and accurate as possible. Specifically, we apply an expert-curated, explicit evolutionary modelling approach to all human protein-coding genes. This approach integrates available experimental information across families of related genes into models that reconstruct the gain and loss of functional characteristics over evolutionary time. The models and the resulting set of 68,667 integrated gene functions cover approximately 82% of human protein-coding genes. The functional repertoire reveals a marked preponderance of molecular regulatory functions, and the models provide insights into the evolutionary origins of human gene functions. We show that our set of descriptions of functions can improve the widely used genomic technique of Gene Ontology enrichment analysis. The experimental evidence for each functional characteristic is recorded, thereby enabling the scientific community to help review and improve the resource, which we have made publicly available.

## Abstract (web summary)

Evolutionary modelling and expert review are applied to integrate experimentally supported knowledge accumulated in the Gene Ontology knowledgebase to create a draft human gene ‘functionome’.

## Main

Human genes are segments of the genome that encode instructions for making molecular machines—primarily proteins but also noncoding RNAs—that perform the functions that create and sustain the human body. Determining the entire functional repertoire of these gene products is vital for understanding human biology and for the treatment of disease. Previously published attempts to comprehensively construct and analyse the entire repertoire of functions encoded by human protein-coding genes were featured in reports of drafts of the human genome sequence in 2001 (refs. ^3,4^). Both publications reported analyses of the set of human protein-coding genes that were state-of-the-art at the time using protein family identification software such as Pfam^5^ and PANTHER^6^ and the nascent Gene Ontology (GO) to define functional classes^7^. These studies reported that a functional characteristic was either known or could be predicted for approximately 40%^4^ and 58%^3^, respectively, of human protein-coding genes. However, these initial studies had several limitations, including that gene function was described at only a high level and there were no traceable links to supporting experimental evidence. As a result, most genes were assigned to a single, broad category of function, and the accuracy of that assignment was not readily verifiable, which made it difficult to build on these analyses as more experimental results became available. Since that time, multiple resources that include human gene functions have been developed^8–10^, including our own work in the GO Consortium^11,12^, but these have not been systematically aimed at a complete, computable representation for human genes.

Here we describe our work to develop a representation of human protein-coding gene functions that is as complete as possible given the currently available data using an approach based on explicit evolutionary modelling^13,14^. This process required the construction of evolutionary models at a large scale: in total, models were constructed for 6,333 phylogenetic trees in the PANTHER database^15,16^ and all available experimental information in the GO knowledgebase. In the resulting representation, the overall function of each human gene is described by a set of multiple functional characteristics (annotations). Each characteristic is represented by a selected term from the GO ontology (a formal ontology of gene functions), is supported by traceable experimental evidence and potentially has a distinct evolutionary history. We have made this set of human gene functions publicly available at https://functionome.geneontology.org.

## Creating a genome-wide set of functions

**Extended Data Fig. 1:**
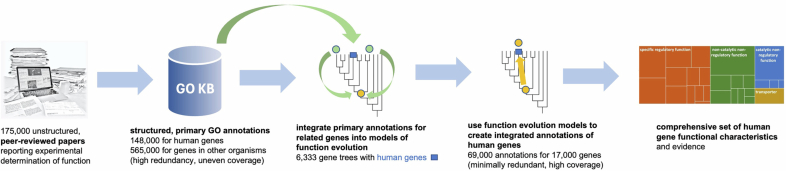
Overview of process for creating the set of human gene functions. First, experimental results from the scientific literature are captured as primary GO annotations, and stored in the GO knowledgebase (GO KB). The next step is phylogenetic integration: a massive corpus of primary annotations for genes in multiple different organisms was integrated using phylogenetic trees that represent the evolutionary relationships between genes. For each gene family tree, selected primary annotations are used to construct an explicit evolutionary model of gains and losses of gene function along branches of the phylogenetic tree, and the evolutionary model is then used to create the integrated PAN-GO annotations for human genes. The set of human gene functions reported here comprises nearly 69,000 integrated annotations.

Our process to create a comprehensive set of human gene functions is shown in Extended Data Fig. 1 and described in detail in the Methods. It relies on expert human curation with extensive computational support. The first step involved the identification of publications that reported experimental findings regarding the functions of genes, from which biocuration scientists (biologists with expertise in data science) created ‘primary GO annotations’. A GO annotation links a gene to a functional characteristic, coupled with the evidence for that assertion. The functional characteristic is selected from the graph of classes (or terms) available from the GO ontology, an information science structure that enables complex experimental findings in biology to be represented in a form amenable for computation. The GO ontology defines three broad categories of functional characteristics: molecular function (MF; functions that the gene product performs at the molecular level); biological process (BP; functions at the level of cellular and organism systems); and cellular component (CC; the cellular structures where the gene product is active). Primary GO annotations currently include findings from more than 175,000 peer-reviewed, published papers, with most of these coming from studies of model organisms. These primary GO annotations are arguably the most extensive and widely used source of functional information about gene function^11,12^. However, they do not constitute a comprehensive representation of human gene functions. First, each primary annotation is limited in scope to a gene functional characteristic that was experimentally demonstrated in a single publication. Consequently, primary annotations are often more reflective of the details of the experiment than the underlying gene function. Moreover, annotations to the same gene can be partially or completely redundant with each other, even if they refer to apparently distinct GO terms (see Methods for examples). Primary annotations are also subject to biases in the published literature; for example, the tendency of studies to focus on only a subset of human genes^17,18^. But perhaps most importantly, direct experimental knowledge of human gene functions remains incomplete. Thus, a comprehensive representation of human gene functions requires leveraging the vast amount of experimental knowledge obtained in a wide range of other organisms, which can provide important information because of the deep evolutionary conservation of many protein-coding genes.

To address these limitations, we implemented a second step to review and integrate the primary GO annotations—for both human and related non-human genes—into a comprehensive, minimally redundant description of human gene functions. This step created a synthesis of the primary GO annotations, analogously to how a review article synthesizes findings from primary research publications. In this approach, called phylogenetic annotation using Gene Ontology (PAN-GO), we performed the following actions: (1) systematically reviewed all functional evidence in the GO knowledgebase for related genes within the evolutionary tree of a gene family; (2) selected a set of maximally informative and independent functional characteristics; and (3) constructed an evolutionary model of how each selected functional characteristic evolved in a gene family (that is, when it arose and, in many cases, was subsequently lost). The evolutionary models were then used to provide integrated PAN-GO annotations for each human gene.

The explicit evolutionary modelling approach represents an advance over previous work to leverage homology information^19,20^, which falls into two broad categories. Methods that use protein families (for example, Pfam^5^ and InterPro2GO^21^) or subfamilies or orthologous groups (for example, PANTHER^15^ or COGs^22^) used in earlier genome-wide functional analyses of the human genome^3,4^ have been updated and regularly expanded. However, they are nonetheless limited to representing functional characteristics that are broadly conserved over an entire family or subfamily and can therefore lack coverage and precision. By contrast, methods that use pairwise identification of homology^23^ or orthology^24,25^ essentially treat each homologous gene pair, and each functional characteristic, in isolation rather than integrating experimental information over multiple related genes. More recently, methods based on deep learning have shown promise^26,27^ but still face similar challenges. In all of these methods, homology relationships at the sequence level are used to make implicit inferences about the evolution of gene functions. To our knowledge, the work presented here is the first to make the evolutionary models explicit at the scale of entire genomes.

## PAN-GO evolutionary modelling process

**Fig. 1:**
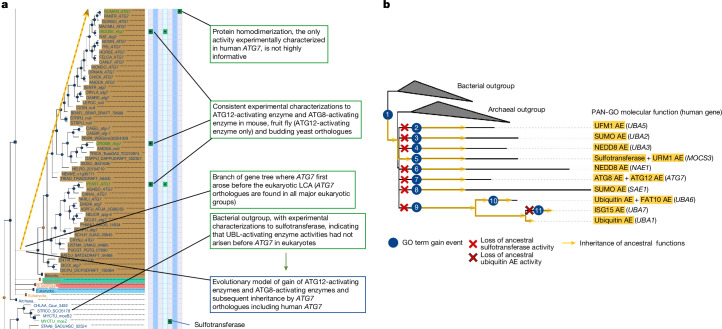
PAN-GO annotation process illustrated using the UAE family. **a**, View of the PAINT software tool (Methods) showing the process of creating a function evolution model for the human *ATG7* gene (top) that integrates function information from related genes. The phylogenetic tree (left) shows the evolutionary relationships between genes found in different organisms. Tree nodes represent speciation events (circles) and gene duplication events (squares); extant genes are labelled with the UniProt five-letter species code^51^ and gene symbol when available. For each extant gene, the sparse experimental function annotations are shown on the right (green squares, each column is a distinct GO class). Information in the gene tree and primary GO annotations (green callouts) is used to construct a parsimonious model for function evolution (bottom callout, dark blue), in which the selected functional characteristics first arose in an ancestral, *ATG7*-like gene. These functions were then transmitted by inheritance to the human *ATG7* gene (dashed yellow arrow). **b**, The PAN-GO evolutionary model and PAN-GO MF annotations for all human genes in the UAE family. Gene duplication events and functional evolution have resulted in ten human genes that serve as activating enzymes (AEs) with different functions at the molecular (shown here), cellular and organism levels (see full model at https://pantree.org/tree/family.jsp?accession=PTHR10953). The PAN-GO function evolution model is shown by circles indicating gains in function, with crosses indicating losses of function and orange arrows indicating inheritance of ancestral function. The LCA of the family had ‘sulfotransferase activity’ (gain labelled 1), which was passed on to the human *MOCS3* gene (arrow leading from 1), but this function was modified in other descendants (losses and gains labelled 2–11) to create the canonical UAEs of varying specificities for different UBLs. For example, human *UBA5* is specific for the UBL called UFM1. Branch lengths represent the numbers of amino-acid substitutions per site. The tree was drawn using the iToL tool^52^.

We first illustrate the modelling process using the example of the ubiquitin-activating enzyme (UAE) family. This family has been well studied in the literature, which enabled us to compare our evolutionary model to previously published findings. The UAE family is found in all kingdoms of life and includes ten human genes. Members of the UAE family can activate a range of ubiquitin-like modifiers (UBLs), small proteins that once activated are attached to other proteins to mark them for regulation. Fig. 1a shows part of the UAE gene family tree in the evolutionary modelling tool called phylogenetic annotation and inference tool (PAINT; see Methods for details), focusing on the clade containing the human *ATG7* gene. The modelling process considered both the gene tree (indicating here the origin of the *ATG7* clade before the last common ancestor (LCA) of eukaryotes) and the sparse experimental knowledge of the functions (primary GO annotations) of the genes in the tree (Fig. 1a, green squares and callouts). The most informative, nonoverlapping set of functional characteristics (GO classes) were selected, and then an evolutionary model was created, specifying the tree branch along which each characteristic arose during evolution (Fig. 1a, bottom callout). The model in Fig. 1 corresponds to the simplest evolutionary model^28–31^ that explains these experimental observations given the evolutionary history in the gene tree. Finally, the PAN-GO annotations for human *ATG7* were assigned by applying the evolutionary model, assuming inheritance (Fig. 1a, dotted line) of the functional characteristics (GO terms) ‘Atg12 activating enzyme activity’ and ‘Atg8 activating enzyme activity’ (GO term labels are indicated in single quotes). In this example, the evidence for human gene function was therefore derived from experiments on related genes in other organisms. For example, the ‘Atg8-activating enzyme activity’ of human *ATG7* is supported by experiments in mouse^32^ and in budding yeast^33^.

Figure 1b shows the PAN-GO model for MFs in the entire UAE family, which contains nine other human genes in addition to *ATG7*, as well as homologous genes from archaea and bacteria. This model demonstrates several important features of the PAN-GO process. First, different members of the same family can have highly different annotations of function. By contrast, the family-based approach used in previous characterizations of human gene functions^3,4^ does not assign a functional characteristic to any genes in the UAE family (see Pfam^5^ PF00899) because the diversity of functions prevents a functional assignment that applies to all members. Second, the model is designed to represent actual evolutionary events, to the degree possible given the reconstructed phylogeny. Because GO classes that describe functions are discrete, we expressed the model of each change in function during evolution as a combination of gains and losses of GO classes. For example, in the branch leading to *ATG7*, the ancestral ‘sulfotransferase activity’ (supported by experimental annotations in the clade of bacterial genes and one clade of eukaryotic genes including human *MOCS3*) evolved into a UBL-activating enzyme activity specific for the ATG12 and ATG8 family of UBLs. In the PAN-GO model, this functional change is modelled as a loss of the GO term ‘sulfotransferase activity’ (as this term is no longer an accurate description of the newly evolved function) and a gain of two GO terms: ‘Atg12 activating enzyme activity’ and ‘Atg8 activating enzyme activity’.

Using only a gene tree and experimental GO annotations, our modelling process constructed a function evolution model that captured the major evolutionary events in this family, which were previously determined through highly labour-intensive detailed studies. In our model, the *MOCS3* clade retains the ancestral ‘sulfotransferase activity’, consistent with previous assertions that MOCS3 provides an evolutionary link to prokaryotic enzymes in this family^34,35^. Although the phylogenetic tree does not reconstruct the order of early gene duplications (shown in Fig. 1b as multiple branches descending from a single ancestral gene), our model does allow us to distinguish the order of gene duplications and functional modifications that occurred more recently to generate the three human genes *UBA1*, *UBA6* and *UBA7*. For these three genes, our model is consistent with a previously published model^36^ in essential details: (1) the ancestral gene was specific for ubiquitin; (2) the duplication leading to *UBA6* pre-dated that leading to *UBA7*; and (3) *UBA7* changed its specificity from ubiquitin to ISG15 (which we modelled as a loss of ‘ubiquitin activating enzyme activity’ and a gain of ‘ISG15 activating enzyme activity’).

## The PAN-GO set of human gene functions

**Extended Data Fig. 2:**
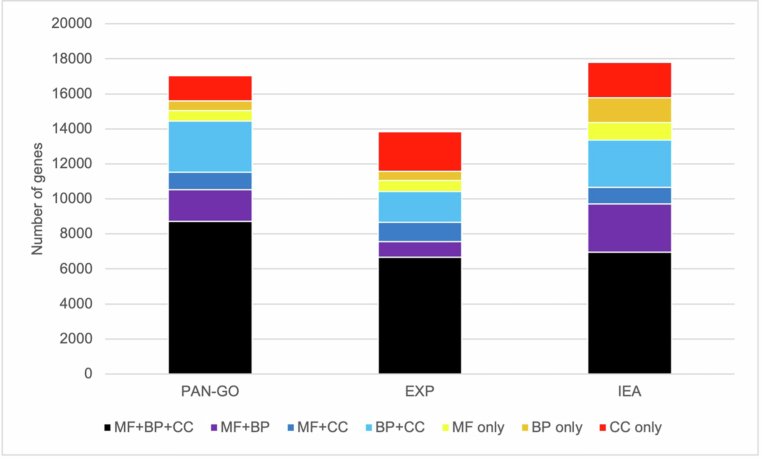
Breadth of annotation coverage of human genes as measured by the number of different aspects of GO (MF, BP and CC) to which a given gene is annotated. For comparison with PAN-GO, two other sources of GO annotations in the GO knowledgebase are shown: primary annotations for human genes (EXP) and computationally predicted annotations (IEA).

**Extended Data Fig. 3:**
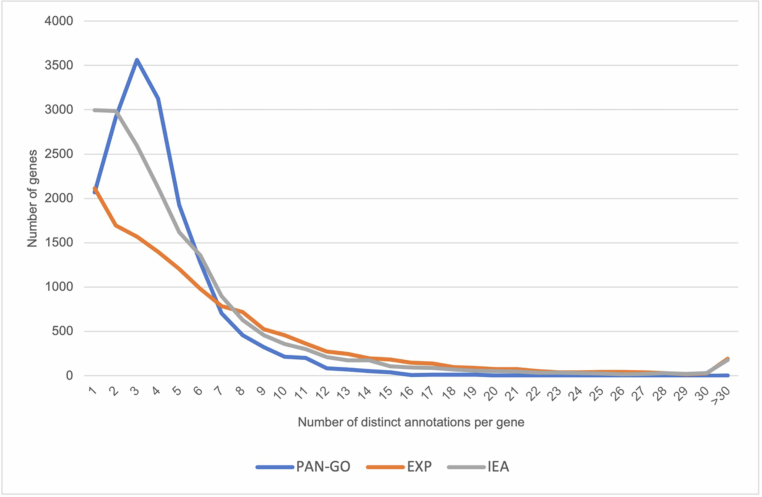
Evenness of annotation coverage as measured by the distribution of distinct GO terms annotated to each human gene. Distributions are shown for PAN-GO annotations, experimental annotations (EXP) and computationally predicted annotations (IEA). Before counting distinct GO terms for genes, we made each set as non-redundant as possible, by removing annotations that are to the same or more general term than another annotation in that set (note that a more general term is implied by the more specific term in the ontology). Direct annotations to ‘protein binding’ (GO:0005515) have also been removed.

The PAN-GO evolutionary models for 6,333 gene families, combined with selected primary GO annotations for a small number (61) of human genes that were not in PANTHER families, resulted in a set of 68,667 integrated GO annotations of function for 17,079 human protein-coding genes (81.9% of the consensus gene set of 20,851 reported by UniProt^37^). PAN-GO annotations covered all three broad categories of the GO ontology, comprising 18,499 MF, 22,022 CC and 28,146 BP annotations. The coverage of genes by GO terms from each broad category is shown in Extended Data Fig. 2, and the distribution of annotations per gene is shown in Extended Data Fig. 3.

To characterize the PAN-GO annotations and to demonstrate their utility, we performed an in-depth comparison of PAN-GO annotations to other available sets of human GO annotations. This included comparisons both to GO annotations from the published literature and to predicted GO annotations using methods that have been extensively tested and reviewed, including several automatic function prediction methods that have been benchmarked in the Critical Assessment of Function Annotation evaluations^20^ or similar benchmarks. Details of the comparison are described in the Supplementary Information, and we summarize here the main findings. First, PAN-GO added 43,206 new annotations for human genes that were not previously present in the set of human primary GO annotations. Of these new annotations, 5,570 refer to related but more specific GO terms and therefore add more functional detail. The majority (37,636), however, are in distinct branches of the ontology and represent functional characteristics that are missing from the set of human experimental annotations. As described in the Methods, we used an established procedure^38^ to estimate a ‘reliability’ of these new annotations between 90–97%. Second, most experimental GO annotations for human genes are excluded from the PAN-GO set. This is due to the selection of largely independent, maximally informative GO terms for inclusion in the evolutionary models. Using datasets from previously published case studies^39^ (Supplementary Information), the PAN-GO set performed better in enrichment analyses than the set of all GO annotations, as PAN-GO avoids a major confounder in these analyses that arises from highly annotated genes^39^. Thus, the PAN-GO set is valuable both for the new annotations added and for the noisy primary annotations removed.

## Experimental evidence for gene functions

**Fig. 2:**
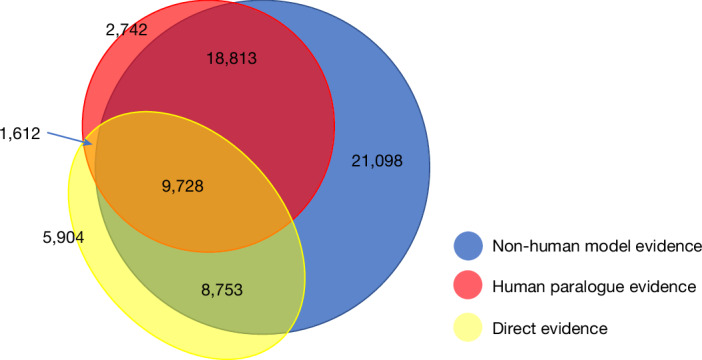
Sources of experimental evidence for PAN-GO annotations. Venn diagram showing the number of PAN-GO human gene annotations according to the source of the experimental evidence used for the PAN-GO annotation.

As described above, each of the 68,667 PAN-GO annotations is supported by one or more lines of experimental evidence. We categorized each line of evidence as direct (based on experiments on a given human gene) or homology-based (based on experiments either on a paralogous human gene or a homologous gene in another organism). Figure 2 shows the distribution of these sources, which considerably overlapped. Notably, only 25,997 (38%) of the PAN-GO annotations were supported by a direct primary annotation (yellow oval in Fig. 2). The remaining 42,670 PAN-GO annotations (62%) were derived from the tree-based homology inference process. Homology inference therefore contributed nearly 1.8 times as many annotations as the direct experimental annotations to the final set of PAN-GO-reviewed human gene annotations. Nearly half of the homology-based annotations (21,098 out of 42,653) were supported by model organism data alone, with no experimental evidence from either the human gene itself or a human paralogue. In addition, nearly all homology-based annotations were supported by model organism data even if they were also supported by human paralogue data (shown in Fig. 2 by the blue area as an almost complete superset of the red area). Even for the human gene annotations that have direct experimental support, there was additional evidence from a model organism over 70% of the time (yellow area overlapping with blue in Fig. 2).

We determined the contributions from each model organism to the human PAN-GO annotations (Extended Data Table 1). The overall trend indicates the contributions depend on both the evolutionary distance to humans and the depth to which an organism has been studied experimentally. Data in the mouse (divergence from human about 80 million years ago) supports around 60% of PAN-GO human annotations, and *Escherichia coli* (divergence about 4,000 million years ago) supports about 3%, a result highlighting that even distantly related organisms can provide insight into human biology.

## The landscape of human gene functions

**Fig. 3:**
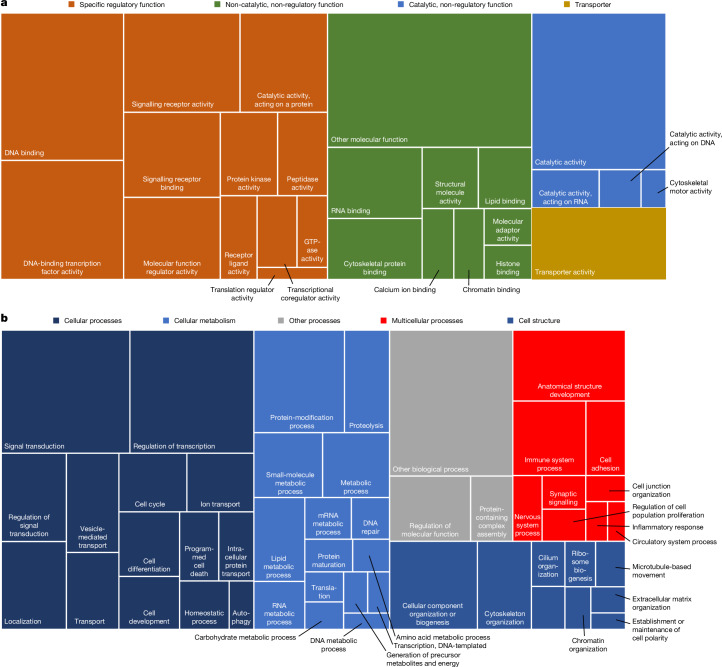
Overview of the set of human protein-coding gene functions categorized by high-level GO classes. **a**, Human genes categorized by MF (activities of encoded proteins at the molecular level) for the 12,117 genes with an MF annotation in PAN-GO. **b**, Human genes categorized by BP (larger system functions to which a protein contributes) for the 13,982 genes with a BP annotation in PAN-GO. For each panel, the areas are proportional to the number of genes in a given functional category. Colours correspond to a few broad categories that do not correspond exactly to GO classes but serve to help organize the GO classes. Note that for the GO classes, some are subcategories of others, and in those cases, annotations are assigned to only the most specific category. For example, a gene annotated with ‘small-molecule metabolic process’ will not be included in the more general ‘metabolic process’. Note also that a gene can be assigned to multiple categories if it has annotations to distinct GO classes that are in different categories.

We used the structure of the GO ontology to group PAN-GO annotations into broad categories to provide insight into the landscape of human protein-coding gene functions (Fig. 3). We analysed two distinct aspects of gene function, the molecular-level functions performed individually by a protein (MF; Fig. 3a) and the systems-level processes performed in conjunction with other proteins (BP; Fig. 3b). For MF, the most noteworthy feature is the preponderance of regulatory functions, which specifically control the activities of other genes and proteins. These were not limited to DNA-binding, gene-specific transcription factors (which was previously reported^3,4,40^) but also included the following other major classes: (1) protein-modifying catalytic activities, including protein kinases and proteases, enzymes that covalently modify other proteins to modulate their functions; (2) regulators of MFs through specific noncovalent-binding interactions; (3) signalling receptors; (4) ligands for receptors; (5) GTPases, primarily large and small G proteins, which are molecular switches that regulate other proteins through binding; and (6) transcriptional co-regulators, most of which modify chromatin to make specific regions of DNA accessible to transcription factors. Together, these classes comprise 5,882 genes, which account for nearly half of the genes with known MFs. A large proportion of human protein-coding genes therefore seem to be involved in the precise control of the performance of other protein functions, a ‘parts list’ with the potential for creating highly complex biological programs. At a higher level of biological organization, Fig. 3b provides an overview of the biological programs (GO BP) that are carried out by multiple genes that function together. These range from programs that occur primarily at the cellular level (for example, biosynthesis, catabolism, cellular structure biogenesis and modification) to those of larger-scale multicellular systems. At the cellular level, the following processes involve the largest number of genes: signalling (that is, the detection, transduction and integration of signals and other stimuli); regulation of transcription (the control of gene expression levels and major responses to signal transduction); cell differentiation (in which a cell develops into a particular type with a specific physiological role); and cytoskeletal organization (the maintenance and change of cell shape). The number of genes implicated in multicellular processes (such as anatomical structure development, immune system processes, and nervous system processes including synaptic signalling) was considerably smaller than for cellular processes. This result is not surprising given that all proteins are expressed in a cell, and thus nearly all genes perform some cellular level function together with other proteins in that cell. By contrast, only a subset will be involved in processes that coordinate the actions among different cells. Moreover, for many multicellular processes, the roles of specific genes are not as well understood as for cellular processes.

## The evolution of human gene functions

**Fig. 4:**
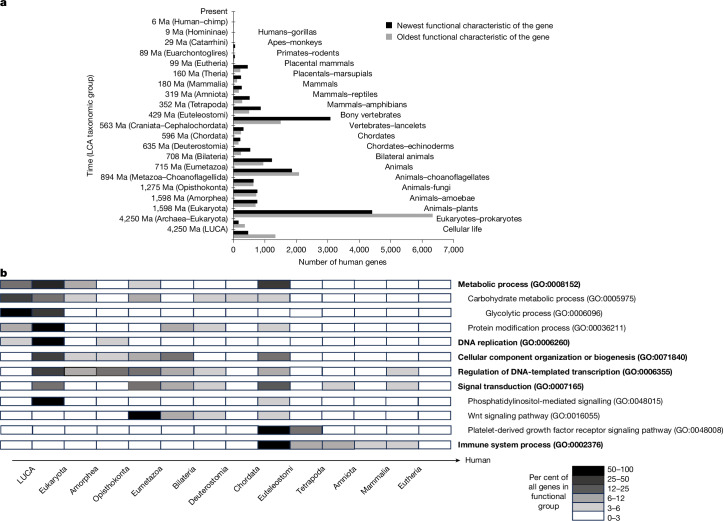
Distribution of the age of human gene functions. Most human gene functions evolved from very distant ancestors. **a**, Distribution of the time periods at which human genes evolved their present-day functions as assessed using two measures: the overall function of a gene (black bars, considering all functional characteristics) and the oldest functional characteristic of a gene (grey bars). Black bars indicate the most recent (newest) functional characteristic to arise in the evolutionary model for that gene, whereas grey bars indicate the age of the most ancient (oldest) functional characteristic among all the functional characteristics for that gene. As shown in Fig. 1, each evolutionary event in our models is mapped to a branch of a gene tree, which represents a period of time separating the LCAs of two different taxonomic groups; the evolution of each functional characteristic is assigned to the corresponding time interval (see Methods for details). As an additional reference, LCAs are expressed in more commonly recognized terms towards the right side. **b**, Age distributions for different types of human gene functions; each time interval is shaded according to the fraction of genes that evolved a given functional type during that interval. Different types of functions display substantially different age distributions, with some basic cellular metabolic functions in humans having remained largely unchanged over billions of years, whereas other groups, such as regulation of transcription and immune processes, have undergone substantial recent evolutionary change. Higher-level functional types are indicated in bold, with more specific subtype names indented below. Taxonomic names are from NCBI Taxonomy^53^, except Amorphea, the group that includes the Amoebozoa and Opisthokonta (fungi and animals). Note that different functional characteristics of the same gene may have evolved at different times. Ma, millions of years ago.

Although we constructed the evolutionary models with the specific aim of creating a comprehensive representation of human gene functions, the models also provide insights about the evolutionary origins of these functions. Fig. 4a shows the distribution of the times at which human gene functions first evolved in terms of the LCAs represented in our models. The distributions show peaks (periods with greater functional innovation) and valleys (less innovation), with four distinguishable peaks: (1) the evolution of eukaryotes; (2) the period spanning the evolution of animals (Eumetazoa) and then bilaterian animals; (3) the period spanning the evolution of vertebrates (Euteleostomi) and then land animals (Tetrapoda); and a smaller peak during (4) the evolution of placental mammals. A similar pattern was previously observed for human gene ages^41^, which is anticipated to correlate to some degree with the evolution of gene functions. However, the gene ages displayed additional, dominant peaks at the extremes of very ancient (before the last universal common ancestor (LUCA) of cellular life) and relatively recent (placental mammals to primates) time periods. This discrepancy may be due in part to the challenges in assigning ages to genes^42^, which shows the importance of considering function directly.

In our models, the majority of human gene functions evolved long ago, and some more than 4 billion years ago (Fig. 4a, black bar below LUCA). Notably, more than half of all human protein-coding genes have inherited a functional characteristic (Fig. 4a, grey bars) that evolved in our distant, single-celled ancestors (before the evolution of multicellularity in animals at least 715 million years ago) and over one-third have not changed in overall function since that time (black bars). By contrast, relatively few human protein-coding gene functions evolved after the LCA of placental mammals (Eutheria) almost 100 million years ago. It is important to note that the functional characteristics in our models are limited to GO terms that have experimental support for a human gene or a related gene and will tend to underestimate true functional changes during evolution. For example, in the PAN-GO model for a cytochrome P450 family (PTHR24300), 18 human genes inherit the GO term ‘xenobiotic metabolic process’ from a common ancestor in which that functional characteristic evolved more than 1 billion years ago. This functional description is correct; however, at a more detailed level not currently captured by GO terms, different human cytochrome P450 families have more recently evolved distinct specificities for different types of xenobiotic chemicals^43^. Nevertheless, the difference between the two distributions in Fig. 4a shows that our models do identify many instances of functional change. If all the functional characteristics of a gene arose at the same time, the grey and black bars would be the same. Therefore, the differences are due to additional functional characteristics that arose later in evolution. For example, of the 1,300 human genes that can trace at least one functional characteristic to a LUCA (bottom grey bar), only 470 (bottom black bar) did not undergo further functional changes. In the period immediately before the LCA of vertebrates 429 million years ago, many genes changed in function (grey bar compared with the black bar). In most cases, these changes are due to the known prominence of gene duplication in the evolution of the vertebrate genome^44,45^. Figure 4a shows how gene duplication manifests in gene function evolution as functional modifications in which a duplicate gains and/or loses some functional characteristics while retaining others.

We used the GO ontology structure to create groups of human genes that share the same type of functional characteristic at various levels of resolution and graphed the distribution of when that characteristic evolved (different genes in the same functional group may have evolved that function during the same interval or different intervals). Figure 4b compares the distributions for selected functional groupings. At a high level of functional grouping (broad groups with many genes), metabolic functions tended to have evolved earlier (most having appeared by the LCA of Eukaryota) and signalling functions substantially later (similar to the overall distribution). Immune system functions appeared relatively recently (primarily in vertebrates through to mammals). At a lower level of grouping, we gained additional resolution. Among metabolic processes, consistent with previous studies^46^, the basic cellular processes of carbohydrate metabolism and especially glycolysis appeared very early in evolution, mostly before LUCA. Human DNA replication functions evolved mostly before the Eukaryotic LCA, but consistent with other phylogenetic studies^47^, our models indicated that the functions of a few components were present in the LUCA, despite the lack of homology between core DNA replication machinery in bacteria and eukaryotes. Most human signalling processes appeared much later, with only a few, such as intracellular phosphatidylinositol-mediated signalling, having evolved before the Eukaryota LCA. Transcriptional regulation showed an additional broad peak of innovation from before the Opisthokonta LCA (the LCA of both animals and fungi) through the Eumetazoa (animal) LCA, when many additional new transcription factor families evolved. Signalling pathways were substantially expanded in animals and then in vertebrates. For example, the WNT signalling pathway evolved before the Eumetazoa LCA (early animal evolution), and the platelet-derived growth factor (PDGF) signalling pathway first evolved before the vertebrate LCA with further modifications before the tetrapod LCA. For human genes with roles in immune system processes, we observed a peak before the vertebrate LCA and a long tail through to the placental mammal LCA, which reflects the evolutionary appearance and further elaboration of the adaptive immune system.

## Discussion

We reported here an initial representation of the functions of human protein-coding genes that aimed to be as complete and accurate as possible: a draft human gene ‘functionome’. Using explicit evolutionary modelling and expert review, we integrated all experimentally supported knowledge accumulated in the GO knowledgebase over the past 25 years for human genes and related genes in highly studied model organisms. Multiple advancements distinguish this functionome from those published previously^3,4^ and from previously available annotations in the GO knowledgebase^11^ (Supplementary Results). Notably, the coverage of human genes is greater, with 82% of genes associated with at least one functional characteristic compared with 40–58% previously reported in the literature, and 67% currently covered by primary GO annotations (Extended Data Fig. 2). Moreover, every PAN-GO functional characteristic has a fully traceable evidence trail, which ultimately links back to the experiments on which it is based (either directly on the human gene, a related gene or both). Finally, PAN-GO annotations represent a synthesis, succinctly summarizing all available primary GO annotations into a nonredundant set of functional characteristics for each gene. We showed that compared with other sources of GO annotations, the number of PAN-GO annotations is relatively consistent across human genes (Extended Data Fig. 3). Furthermore, using PAN-GO directly in enrichment analyses reduced a major source of bias that arises from highly annotated genes^39^ (see the section ‘Comparison of gene set enrichment analysis results’ in the Supplementary Results). Unlike other sources of GO annotations, in PAN-GO each selected GO term is designed to represent a distinct functional characteristic, and the annotation set is therefore minimally redundant. This feature may be unfamiliar to many GO knowledgebase users, who may assume that more annotations are always better and that distinct annotations always represent distinct functions. This property may also make the PAN-GO annotation set useful as a training set for machine learning in the prediction of gene functions.

The evolutionary modelling approach enabled us to draw some preliminary conclusions about the evolution of human gene functions. We found that most human gene functions were inherited from very ancient ancestors, before the evolution of the first multicellular animals. The actual fraction may be even larger, as phylogenetic approaches in general have been shown to underestimate gene ages in protein families with highly diverged sequences, for which homology cannot be reliably established on the basis of protein sequence similarity^48^. However, we probably underestimated the number of recent, relatively fine-grained functional changes that have occurred during evolution owing to lack of experimental data and insufficient precision of GO terms. Our models also showed that different types of functions appeared and were then further elaborated with additional functional components during different time periods, which led to a range of distinct patterns of evolution.

The functional information from non-human genes was crucial for achieving the high coverage and specificity of PAN-GO annotations. However, it also showed that even with the inclusion of extensive knowledge from studies of model organisms, the current collective knowledge of the human functionome remains incomplete. In the PAN-GO annotation set, roughly 30% of human genes have either no annotations or annotations to just one aspect of the GO ontology, similar to another recent estimate of the ‘unknowme’^49^. We anticipate that the PAN-GO annotations will help identify gaps in our knowledge that can be filled by new experiments and by the inclusion of existing publications that are not yet in the GO knowledgebase. We encourage the community to review the human gene functions in which they have expertise and submit suggested publications that should be added to the GO knowledgebase and incorporated in the PAN-GO annotation set. We also anticipate that additional functional information for poorly characterized human genes will be added from high-throughput phenotyping of gene knockouts in human cells and tissues (https://morphic.bio) and mouse embryos^50^. The comprehensive set of human gene functions we present here should therefore be viewed not as an end point but as a snapshot that will be continually refined and expanded over the coming years, building on the work of a large international community of experimental, computational and biocuration scientists.

## Methods

### Primary GO annotations

The process for creating GO primary (experimental) annotations from the published literature has been previously described in detail^54^. New annotations from additional publications are added at the rate of approximately 4,000 per month, and some annotations, if they have been superseded in light of new experimental results or updates in the biological representation captured in the ontology, are revised or removed. Scientific publications used to support experimental GO annotations are labelled with a PubMed LinkOut^55^ whenever possible and can be retrieved at https://pubmed.ncbi.nlm.nih.gov/?term=loprovGeneOntol%5bSB%5d. A small number of additional publications are not indexed by PubMed. Our analyses used the ontology and annotations from the GO knowledgebase release 22-03-2022 (https://release.geneontology.org/2022-03-22/index.html, 10.5281/zenodo.6399963). There were 713,330 primary annotations, including 147,872 annotations to human genes and 565,458 to genes in other organisms. For all annotation counts, we excluded direct annotations to the class ‘protein binding’, as these statements represent observed interactions but are not descriptions of function in the same sense as other GO annotations^56^, and are therefore not considered for inclusion in the PAN-GO set.

### Overview of the evolutionary modelling approach

Our approach^13^ brings together all experimentally supported GO annotations for all members of a gene family, in the context of a phylogenetic tree representing how those genes are related, to generate a model of the evolutionary process by which the members obtained the functions they now possess. This is a longstanding, standard method for reconstructing the evolution of traits or characters that is commonly applied to species^28–31^. Here we applied a similar approach to trees of genes rather than species and to functional characteristics rather than phenotypic characters. However, modelling gene functional characteristics involved the major additional challenge that the experimental data are sparse and highly unevenly distributed. Genes have been studied to widely varying degrees depending on scientific and medical interest, and this interest has been largely concentrated on genes in humans and a handful of model organisms. To address this challenge, we also use many other pieces of evidence, such as protein domain structure, known active-site residues, free-text function descriptions from the UniProtKB/Swiss-Prot knowledgebase^37^, among others.

For each gene family, we generated an evolutionary model that specifies how each functional characteristic, represented by a GO class, was gained or lost during evolution. Specifically, we describe the evolution of function in terms of three types of event: root, gain and loss. A root event is defined as a GO class that is inferred to have been present in the LCA of the protein family. A gain event is defined as a GO class that was not (or cannot be confidently inferred to be) present in the LCA of the entire family, but arose later along a specific branch of the tree. A loss event is defined as a GO class that had arisen earlier (through a root or gain event) but was subsequently lost along a specific subbranch of the tree (that is, in some but not all descendants of the original root or gain).

Every root or gain event must be supported by direct experimental evidence in at least one, but often multiple, of the descendants of the root or selected branch of the tree. As a result, each event is based on a combination of traceable experimental evidence and curator inference of the point in evolution (the root or a specific branch in the tree) at which this function first appeared. The Evidence and Conclusion Ontology (ECO)^57^ evidence code IBD (ECO:0000319 ‘inferred from biological descendant’) was used to denote this type of evidence, and all genes with experimental evidence are stored as metadata to provide a traceable evidence trail. Loss events prevent GO classes from being inherited by specific subclades that descend from a gain event; the evidence used for loss events is described in more detail below.

The evolutionary model for the family was then used to create inferred annotations for each family member based on inheritance from ancestors in the tree: a GO class is inherited by all children of a root or gain event for that class unless a loss of that same class is encountered along the path in the tree. All family members will therefore receive the same GO annotations if the family has only root events, but different annotations if there are any gain or loss events along specific internal branches of the tree. These inferred annotations comprise the set of human gene functions we describe here and can be identified in the GO knowledgebase by the ECO code ‘inferred from biological ancestor’ (IBA) (ECO:0000318). Each IBA annotation also includes the following metadata for providing a traceable evidence trail: (1) the persistent identifier of the tree node from which the annotation was inherited (the root node or terminal node of the annotated gain branch); and (2) the source of the experimental data used to support the root or gain event.

### PAN-GO evolutionary modelling process

**Extended Data Fig. 4:**
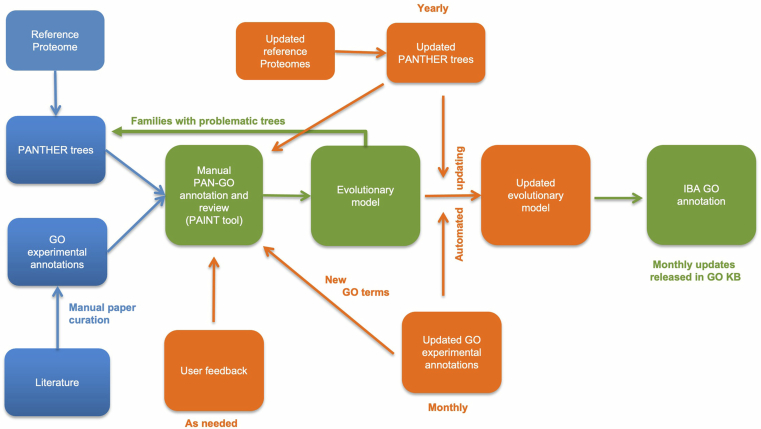
Process for creating, updating and releasing evolutionary models and PAN-GO (IBA) annotations derived from the models. The central task is the software-assisted process of PAN-GO annotation and review using the PAINT tool. PAINT integrates the primary GO experimental annotations with the PANTHER trees built from UniProt Reference Proteomes (blue squares), allowing curators to construct an evolutionary model of each gene family, which is used to produce annotations in family members (green). Updates, both automated and curator-reviewed (orange squares), are made at each GO knowledgebase release to reflect changes in the underlying data (ontology and annotations), and upon release of new PANTHER versions.

A more detailed description of the process of producing and updating PAN-GO annotations is shown in Extended Data Fig. 4. The process includes manual construction of an evolutionary model for each family, using as input PANTHER phylogenetic trees and primary GO annotations. Both automated and manual updates are performed in response to user feedback, changes in biological knowledge in the ontology, changes in primary annotations and changes in PANTHER tree topology. Updated PAN-GO gene annotations (IBA) are generated monthly from these updated models. The different steps leading to the final PAN-GO gene annotations are described in this section.

### Phylogenetic trees

The gene trees were obtained from the PANTHER knowledgebase^15^. The PAN-GO annotation set presented here was generated using v.15.0 of the knowledgebase, released in 2020. Trees were constructed using the GIGA tree reconstruction algorithm^58^ for protein-coding genes in 142 organisms that span the tree of life, but the selection of organisms (https://pantherdb.org/panther/speciesTree.jsp) was biased with the aim of reconstructing genome evolution in humans and well-studied model organisms. The trees were fully reconciled with the known species tree, and all nodes were annotated by event type (speciation, gene duplication and horizontal gene transfer) and the common ancestor species or clade for speciation nodes. Each tree has an associated protein sequence alignment that was used to reconstruct the phylogeny. Protein sequences were obtained from the UniProt Reference Proteomes resource^37^, which selects one canonical protein sequence per protein coding gene in each genome.

### Creating curated models of function evolution

To implement the PAN-GO process, we created a specific software tool for manual curation of function evolution models, which we call PAINT^13^. The PAINT user interface provides an integrated view of the phylogenetic tree, a matrix of experimental GO annotations structured by ontology relationships, a multiple sequence alignment annotated with functional sites from UniProt/Swiss-Prot records^37^ and domains from the Pfam resource^5^. It also displays brief free-text descriptions of the protein products of each gene in the tree, protein names and links to pages in knowledgebases including UniProt/Swiss-Prot and model organism databases. PAINT enables expert biocuration scientists to transform the input information, a phylogenetic tree with experimental GO annotations on terminal (leaf) nodes of the tree, into an output evolutionary model as described above. The specific guidelines for constructing models of function evolution in a protein family, to promote consistency and reproducibility of the evolutionary models, are detailed at https://wiki.geneontology.org/PAINT_User_Guide. Curators also meet regularly to review sample families from each curator, to review and to cross-check the evolutionary models. The evolutionary models are saved to a relational database and can be accessed and viewed at https://pantree.org. The PAN-GO annotations derived from the models are exported in Gene Annotation Format (GAF) (https://geneontology.org/docs/go-annotation-file-gaf-format-2.2/) and deposited in the GO knowledgebase. They are also included in the data distributed by providers of GO annotations such as UniProt-GOA^59^. These annotations are labelled with the evidence code IBA, and contain metadata with details of the evidence or provenance, including the curated tree node from which it inherited its function (represented as a stable PANTHER tree node identifier) and the genes providing the original experimental evidence. Source code for the PAINT tool is available at GitHub (https://github.com/pantherdb/db-PAINT).

#### Inspection of the phylogenetic trees

The first step of the PAN-GO curation process consists of the analysis of the structure of the phylogenetic tree to gather clues about the evolution of the family. Speciation, duplication and horizontal transfer events are closely considered. Speciation events define the age of the family and the taxonomic distribution of related genes in different clades. This information helps guide the choice of GO classes based on the functions known to occur in the species present in a tree or subtree. A more ancient ancestor (which generally leads to a wider species distribution) may lead to more conservative annotations owing to uncertainty in reconstructing ancient functions. The tree can also provide other important clues for identifying functional evolution events. Duplication events are examined closely as these events often lead to gain and/or loss of functions. Horizontal gene transfers, which include some eukaryotic mitochondrial or plastid genes with origins in ancestral prokaryotic endosymbionts, are also carefully evaluated, as functional characteristics of a transferred gene may have been modified after transfer.

#### Application of taxonomic restrictions

Because of the high diversity of living organisms, it is not possible to cover all species with a taxon-neutral ontology and there are inherent taxon specificities in many branches of the GO ontology. An iconic example is the cellular component ‘mitochondrion’, which is specific to eukaryotes. Explicit formalization of taxon constraints^60^ are used to avoid taxon-inappropriate annotations. The PAINT curation tool highlights any inconsistencies between taxon constraints and annotations when constructing an evolutionary model.

#### Analysis of the experimental evidence

The analysis of all the experimental data available enables the selection of the most relevant classes that will be used in the evolutionary model for a gene family. An essential indicator is the consistency of the MF, BP and CC classes associated with the various members across species represented in the tree. If the annotations in a clade of related genes are consistent, they are likely to have all inherited those aspects of function from their LCA, which suggests that those functions evolved before the LCA. If they are inconsistent, a curator attempts to identify consistent subclades that evolved a different function, or gained or lost a function. Assessing consistency among GO classes that are not explicitly related in the ontology structure is challenging and often requires deep biological knowledge on the part of the curator. To decide which classes are appropriate to be associated with members of a protein family, the PAN-GO curators use additional sources: they can review the content of model organism databases or UniProtKB/Swiss-Prot (https://www.uniprot.org) through direct links provided by the PAINT tool. Curators often assess additional references to confirm or invalidate certain data. Finally, the presence of particular predicted sites and domains (active sites, transmembrane regions or protein domains) may lend more support for specific functions having evolved along particular branches in the tree.

### Selection of the most informative annotations

In principle, PAN-GO curation could have resulted in an evolutionary root or gain event in the tree for every GO class that was annotated to at least one family member from experimental evidence. In practice, however, there is often considerable redundancy and overlap between these GO classes, and not all terms represent actually distinct functional characteristics. Consequently, the PAN-GO curation process is selective. We provide some examples below. To provide a quantitative estimate of the selectivity, we counted, for each family, the number of nonredundant function classes (that is, excluding annotations to more general classes in the ontology) that were available to a curator; these were all the classes that could have been used in the evolutionary model for the family. We then calculated the number of classes actually used in the evolutionary model for each aspect of the ontology. Extended Data Table 2 shows the average of these values over all families. On average, only 24%, 28% and 13% of the experimentally annotated MF, CC and BP GO classes, respectively, were annotated to root or gain events during the phylogenetic curation process. In general, this high selectivity is due to the integrative aspect of the process: all experimental GO annotations for all family members are considered as a whole. By contrast, an experimental GO annotation is designed to capture a specific finding from experiments reported in a single publication. As a result, a PAN-GO curator can select the most informative GO classes among the experimental annotations and recognize when different experimental annotations are likely related to the same underlying function. Often, functionally related terms are also related in the ontology (the PAINT tool groups together hierarchically related terms to facilitate the selection process). Curators can then distinguish such apparent functional differences from actual functional differences among family members. Extended Data Table 2 shows that the PAN-GO curation process results in selection of a relatively small fraction of GO biological process classes compared with the other aspects of the GO ontology. This is due in part to the complexity of the biological process branch of the ontology (around 30,000 classes versus <10,000 each for MF and CC), and partly due to less stringent criteria for involvement in a process versus the other aspects. Many of the excluded classes are either related but less informative classes or downstream effects of the primary functions of the gene, such as peripheral functions or phenotypes and readouts that represent consequences of a gene’s function but not accurate descriptions of the function itself.

**Extended Data Fig. 5:**
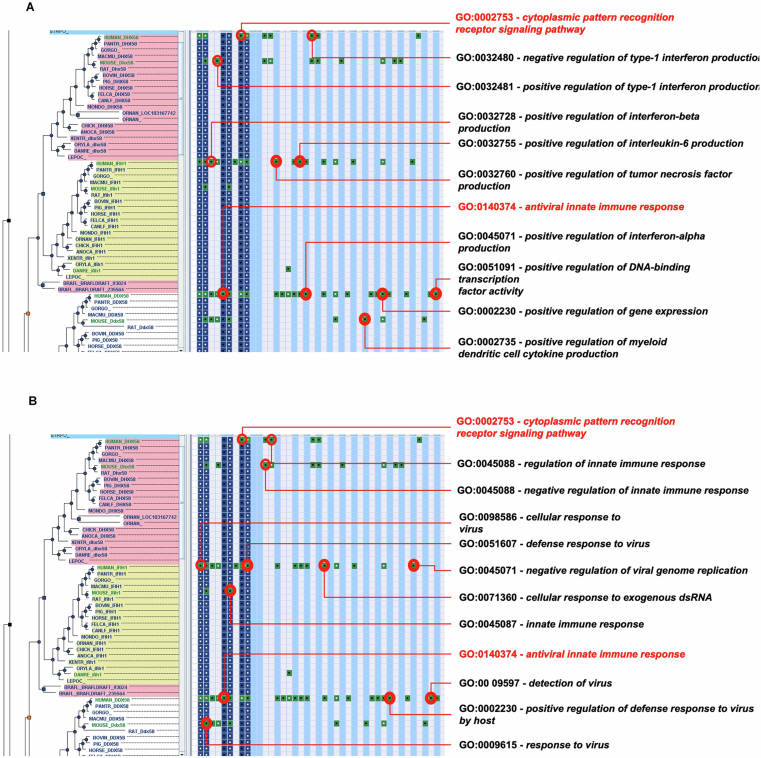
Selection of GO classes (functional characteristics) for evolutionary modeling from among available classes with experimental evidence (primary GO annotations). This figure shows part of the tree corresponding to PANTHER family PTHR14074 (which includes genes involved in recognition of viruses and other pathogens) in the PAINT tool. Out of over 40 BP classes associated with this family through primary GO annotations, only two have been selected for the evolutionary model: ‘antiviral innate immune response’ and ‘cytoplasmic pattern recognition receptor signaling pathway’ (red text). The other classes (black text) correspond to peripheral processes or phenotypes (A), or are related classes, parent or child classes of the most relevant classes (B) and have not been selected for the evolutionary model. Green squares indicate primary GO annotations for the gene in that position of the tree, and red circles highlight different BP classes annotated to the members of this family during GO primary annotation.

An example of BP class selection is shown in Extended Data Fig. 5a: the regulation of production of various interleukins and transcription of downstream targets are observations (experimental readouts) for the ‘cytoplasmic pattern recognition receptor signalling pathway’. There are several reasons that primary annotations for the same underlying function often use related, but not identical, GO classes: primary annotation is spread out in both space and time, and each species is often treated by a different curator. This is compounded by the fact that some functional characteristics (GO terms) are important in a few species but too specific for inclusion in the evolutionary model. Moreover, the authors of the articles from which the primary annotations are drawn use widely varying terminology. Primary GO annotations that are only supported by data from large-scale experiments (most typically, cellular localization) or annotations inconsistent with all other data available for the family are set aside until there is strong support by other annotations.

In many cases, parent and child classes (indicating less specific, representations of a functional characteristic) are both used for primary annotation throughout the families, but only the most relevant ones are selected in the PAN-GO process (Extended Data Fig. 5b): the GO terms ‘regulation of innate immune response’ and ‘cellular response to virus’ are more general classes for the concept ‘antiviral innate immune response’, which is more representative of the function of genes in the family. It is the integrated analysis of the family and its primary annotation that enabled the PAN-GO curator to select the most appropriate class (or classes) to include in the evolutionary model.

As in the ‘three blind men and the elephant’ parable, primary annotations, which describe individual experimental observations, are generally correct but sometimes only tell part of the story. The goal of the PAN-GO curation is to provide a more integrated picture whenever possible while still providing a comprehensive set of GO function annotations.

#### Capturing loss of function and preventing inheritance of low-confidence annotations

Loss of function is based on specific types of evidence when available. In some cases, negative primary GO annotations (indicated by the NOT qualifier) are available, and in this case the loss event (like root and gain events) uses the IBD evidence code. In other cases, when important residues or domains are known to be required for the function, multiple sequence alignments can reveal the absence of these important features in some branches and provide evidence for the loss of function; these are denoted with the ‘inferred from known residues’ (IKR) (ECO:0000320) evidence code. The loss of function due to mutations in specific amino acids such as active site residues is well characterized in the literature for some families (for example, PTHR24418, non-receptor protein kinase family). For families with relatively well-studied genes, it is often possible to infer that a lack of corroborating GO annotations suggests that the function has been lost; in these cases, curators check the UniProtKB/Swiss-Prot knowledgebase as well as the literature to increase the confidence of such inferences. In less well-studied families (that is, with sparse experimental GO annotations), curators may decide to introduce a loss (particularly after gene duplication) to avoid false-positive annotations. These events are denoted by the ‘inferred from rapid divergence’ (IRD) (ECO:0000321) evidence code. The main purpose of this step is to remain conservative in the PAN-GO inference process to ensure the high quality of the annotation set produced. It should be noted that loss events labelled with IBD or IKR result in negative GO annotations (indicating that a gene does not possess a given functional characteristic), and these annotations are available in the GO knowledgebase. However, for clarity, we do not include negative annotations in the PAN-GO set of human gene functions available at https://functionome.geneontology.org, and these appear only in the evolutionary models.

#### Annotations for genes that were not in a PANTHER family

There are 994 human genes that are not currently in a PANTHER family, and these mainly encode short proteins, many of which do not exhibit clear evolutionary conservation. Only 114 of these genes had primary annotations. For 61 of these genes, we were able to select informative primary annotations and included them in the PAN-GO set of human gene functions.

### Staying current with evolving knowledge

As the GO ontology and primary gene annotations are constantly being expanded and revised in response to new experimental data and interpretation, the PAN-GO process includes an automated updating and publishing step after each new GO knowledgebase release (approximately monthly) or each new PANTHER release (yearly). In addition, issues identified by feedback from GO curators and the wider GO user community lead to manual review of the ancestral annotations (or, much less commonly, trees) as appropriate. The PAN-GO project has developed an extensive software suite to support these updates and improvements.

#### Addressing changes to GO classes and annotations

The monthly automated updating step after each new GO knowledgebase release handles any required action due to changes in the ontology classes (terms) or experimental GO annotations that were used as evidence for the functional evolution events in the evolutionary model. These actions include updates for obsolete and merged classes, and the removal of any annotation no longer supported by experimental data or failing taxon restrictions.

Evolutionary models are also updated according to the availability of new experimental data and subsequent primary GO annotations, as new classes and new annotations cannot be integrated automatically but go through manual analysis of the experimental evidence. For instance, during the complete review of the ontology associated with transcription, the class ‘histone chaperone activity’ was created, and primary annotations were revised. This new class was used to update the evolutionary models of applicable PANTHER families such as PTHR21315 or PTHR12040.

#### Addressing updates to the topology of phylogenetic trees

The phylogenetic trees are updated after release of new PANTHER versions, based on the annual release of the protein sequence data from the UniProt Reference Proteomes and Quest for Orthologs efforts^61^. PAN-GO evolutionary models refer directly to stable tree-node identifiers; that is, each gain and loss event is associated with the identifier for the terminal node of the branch along which the event occurred. As tree-node identifiers are retained between PANTHER versions whenever possible, the PAN-GO annotations for those branches are retained in the newer version of PANTHER trees. However, improvements in tree reconstruction algorithms and the addition of more species sometimes lead to modifications of the family structure: some families can be split into several smaller families or merged into a single, larger family. Consequently, some branches can move from one family to another or be lost. When this happens to a branch that was annotated in a PAN-GO evolutionary model, a ‘require review’ notification is added to the affected families, and curators review and revise the evolutionary models when necessary.

#### Addressing user feedback

Extensive feedback from experts from several model organism databases permitted the addition of an extra layer of quality control to the PAN-GO evolutionary models. Feedback is handled through the GO annotation issue tracker in GitHub (https://github.com/geneontology/go-annotation/labels/PAINT%20annotation). The two largest contributors of feedback tickets have been PomBase, the scientific resource for *Schizosaccharomyces pombe* (fission yeast) (https://www.pombase.org/)^62^, with nearly 600 update requests, and FlyBase, the scientific resource for *Drosophila melanogaster* (fruit fly) (https://flybase.org/)^63^, with over 200 update requests, over a 7 year period. The genomes of *Drosophila* species contain many traces of more or less ancient duplication events, which also enable a better understanding of these events in the whole phylogenetic tree and contribute to improving our evolutionary models of gain or loss of functions^64^. The other resources in the GO Consortium, including model organism databases and UniProtKB, also contributed to the validation of the annotations (total of 100 update requests).

### Analysis methods

#### Accessing and using the human PAN-GO annotations

The PAN-GO annotations used for the analyses presented here can be downloaded at https://functionome.geneontology.org/download/functionome_release.gaf.gz.

#### Estimating the reliability of PAN-GO annotations

There is no absolute source of truth that enabled us to assess the correctness of GO annotations. To address this problem, a surrogate measure called ‘reliability’, which can be calculated for GO annotations, has been previously proposed^38^. This measure takes advantage of the fact that GO annotations are being added and removed over time, and they can be compared at different time points to calculate the reliability of older annotations. Specifically, if an experimental annotation is later added to the GO knowledgebase that is to the same or more specific term than an older annotation, the older annotation is considered to be confirmed. Conversely, if an experimental annotation is later added to the GO knowledgebase that uses the NOT qualifier (indicating that a gene has been shown NOT to have that functional characteristic) and is either the same or less specific than the older annotation, the older annotation is considered to be rejected. Because NOT annotations are rare in the GO knowledgebase, the number of rejected annotations is low in practice, thereby leading to an inflated reliability. The previous study^38^ suggested that another property could be calculated, the number of older annotations that were later removed, based on the assumption that they were later judged to be incorrect. They then defined reliability as:

1 $${\rm{Reliability}}={N}_{{\rm{confirmed}}}/({N}_{{\rm{confirmed}}}+{N}_{{\rm{rejected}}}+{N}_{{\rm{removed}}})$$

where *N*_confirmed_ is the number of GO annotations present in an older version (at time point *t*_0_) of an annotation set, which were later confirmed before time point *t*_1_, *N*_rejected_ is the number of GO annotations present at time *t*_0_ that were rejected between time points *t*_0_ and *t*_1_, and *N*_removed_ is the number that were removed between time *t*_0_ and *t*_1_.

Using this method, we calculated the reliability of PAN-GO annotations. We first gathered all primary annotations made between October 2019 and March 2022 from the GO knowledgebase using the date stamp on each annotation. We then compared them with the PAN-GO annotations in the October 2019 release of the GO knowledgebase. The comparison included 11,102 new primary annotations and 21,145 PAN-GO annotations for the same set of 4,007 human genes. If the GO class from the new primary annotation is the same or more specific as that of a PAN-GO annotation, the PAN-GO annotation is considered to be confirmed. By this definition, 2,354 PAN-GO annotations for 1,608 genes were confirmed. Extended Data Table 3 shows the breakdown of the confirming primary annotations by evidence code; most of these derive from direct assays on a specific gene product (IDA), and only 29 were from high-throughput studies (HDA).

Of the new experimental annotations, there were 54 negative (NOT qualifier) annotations, of which only three disagreed with PAN-GO annotations. After reviewing these three negative annotations, we found that one was specific to one protein isoform but not the canonical protein encoded by the gene (so the PAN-GO annotation is correct), and the remaining two were to the same transporter gene and refer to zinc as a substrate (SLC30A10 NOT ‘zinc ion transmembrane transporter activity’, and SLC30A10 NOT ‘intracellular zinc ion homeostasis’). However, other papers (supporting other primary GO annotations) have demonstrated these same functions for SLC30A10, and therefore confirm the PAN-GO annotations. As a result, there were 0 negative GO annotations that can be considered to reject PAN-GO annotations. We recognize that 54 negative annotations is a small sample, which will underestimate the actual PAN-GO error rate. Following the previously described method^38^, we also examined the PAN-GO annotations that were present in our October 2019 release but later removed. We found 4,809 PAN-GO annotations had been removed, but in most cases, annotations were removed owing to redundancy with another, more informative PAN-GO annotation (fine-tuning of the annotation set) and not because of an error. To estimate an error rate, we reviewed a random sample of 500 removed annotations and categorized each one as correct but not meeting PAN-GO selection criteria (fine-tuning of selected annotations for modelling), incorrect (selection in the evolutionary model of an experimental annotation that is actually incorrect) or uncertain (demonstrated in a homologue but possibly incorrect for the annotated human gene). We found that 7 (1.4%) were incorrect and 20 (4%) were uncertain. Assuming these percentages approximately hold for the entire set of removed annotations, we estimated that between 67 (removed because they were incorrect, 4,809 × 1.4%) and 260 (removed because they were either incorrect or uncertain, 4,809 × 5.4%) were removed because of errors. This would give a reliability (equation (1) above) of PAN-GO annotations between 90% (260/(2,354 + 260)) and 97% (67/(2,354 + 67)).

One example of a clearly incorrect PAN-GO annotation was found within the carnitine *O*-acyltransferase family (PTHR22589). CPT1C, in contrast to the CPT1A and CPT1B paralogues, does not have ‘carnitine *O*-palmitoyltransferase activity’ in mitochondria, but localizes in the endoplasmic reticulum where it shows ‘palmitoyl-(protein) hydrolase activity’^65,66^. This type of incorrect inference of functional conservation through ancient duplication events, and therefore errors in evolutionary modelling, is one of the most common errors we found during our review. When such errors are discovered, the PAN-GO evolutionary model is updated to correct the error.

A relatively frequent case of important fine-tuning of PAN-GO annotations relates to the sometimes subtle difference between a GO term for a BP and the corresponding GO term for regulation of that process. Frequently, the primary annotation derived from an experiment, often based on the effects of a genetic manipulation such as a deletion, uses the regulatory term. Other experiments, however, may show that the protein in question is directly involved in the process (resulting in an annotation to the process itself rather than its regulation). Several PAN-GO annotations were updated (5 in our sample of 500) to consistently reflect either involvement in, versus regulation of, a particular BP. Other common updates were due to inconsistencies in the primary annotations for enzyme complexes to the GO term ‘complex assembly’ (10 in our random sample of 500), which we consider to be fine-tuning as they are correct even if not highly informative.

#### Broad functional categories on the PAN-GO website

To facilitate browsing of the PAN-GO annotations, and for visualizing the landscape of human gene functions in Fig. 3, we mapped each annotation to a set of selected, relatively high-level GO categories. Broad functional categories were taken from the generic GO subset, which is available at https://release.geneontology.org/2022-07-01/ontology/subsets/goslim_generic.obo. Note that these are categories of annotations, not genes, so a gene annotated to multiple distinct GO terms may appear in multiple categories. Note also that some of these broad categories are subcategories of others; in this case, a gene was assigned only to the more specific subcategory, and not the more general category, to minimize the overlap between categories and therefore facilitate visualization and browsing.

#### PAN-GO annotation browser

We developed a simple web-based tool for exploring the set of human gene functions, including links to all experimental evidence and phylogenetic trees. It is implemented using ElasticSearch and is available at https://functionome.geneontology.org/. Code is available from GitHub (https://github.com/pantherdb/pango).

#### Contributions of experimental evidence from model organism annotations

Primary GO annotations (supported by published experimental evidence) are used for all PAN-GO annotations. We characterized this evidence in detail for each model organism (Extended Data Table 1). Column 2 reports the number of PAN-GO annotations that are supported by one or more publications with experimental evidence for function of a gene in that organism. Evidence obtained from experiments on human genes is divided into two rows: one for direct evidence for a given gene and one for evidence for a related (paralogous) human gene. Column 3 reports the number of PAN-GO annotations supported only by experimental evidence for homologous genes (that is, it excludes any PAN-GO annotations that have direct experimental evidence for the human gene). These annotations were inferred from other human paralogues or non-human homologues, but have not yet been experimentally confirmed. Column 4 counts PAN-GO annotations that are based on non-human experimental data only. Column 5 counts PAN-GO annotations that are based on evidence from only one species. Column 6 counts all experimental annotations in each organism that could potentially be used as literature evidence for human PAN-GO annotations.

#### Evolution of gene functions

For each PAN-GO annotation, we retrieved the branch of the evolutionary tree that was modelled as having gained that functional characteristic, representing when that characteristic first evolved in an ancestor of a human gene. Because the phylogenetic approach defines ancestors in terms of LCAs of extant species, our evolutionary model specifies the interval between two of these LCAs, during which the functional characteristic evolved. The approximate dates for each of these LCAs has been determined^67^, so we could convert the LCA interval to a time interval. For instance, if a gene function characteristic now found in a human gene first appeared along the branch leading from the LCA of Eukaryota and Archaea (around 4,250 million years ago) to the LCA of plants and animals (the LCA of Eukaryota, about 1,598 million years ago), then the function first evolved between 4,250 and 1,598 million years ago, and was then transmitted unchanged from parent to child for at least 1.6 billion years all the way to modern humans.

### Reporting summary

Further information on research design is available in the Nature Portfolio Reporting Summary linked to this article.

## Online content

Any methods, additional references, Nature Portfolio reporting summaries, source data, extended data, supplementary information, acknowledgements, peer review information; details of author contributions and competing interests; and statements of data and code availability are available at 10.1038/s41586-025-08592-0.

## Supplementary information

Supplementary Information This file (PDF format) contains comparisons between PAN-GO human function annotations and GO annotations from other sources, including comparisons of GO enrichment analysis results. It comprises Supplementary Results, Supplementary Fig. 1 and Supplementary Tables 1–8.

Reporting Summary

## Figures and Tables

**Extended Data Table 1:**
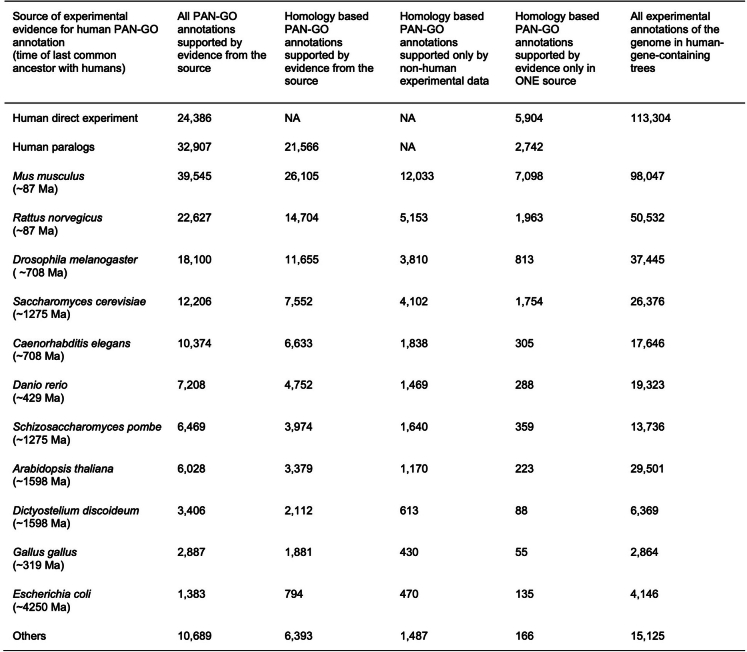
Sources of experimental evidence for human PAN-GO annotations

Divergence times (Ma, million years ago) from humans are taken from the TimeTree resource^67^.

**Extended Data Table 2:**
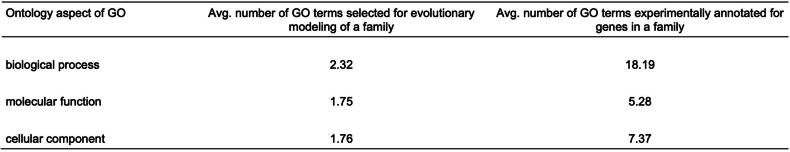
Average number of GO classes selected for evolutionary modeling, compared to total number of experimentally annotated GO classes in each family

**Extended Data Table 3:**
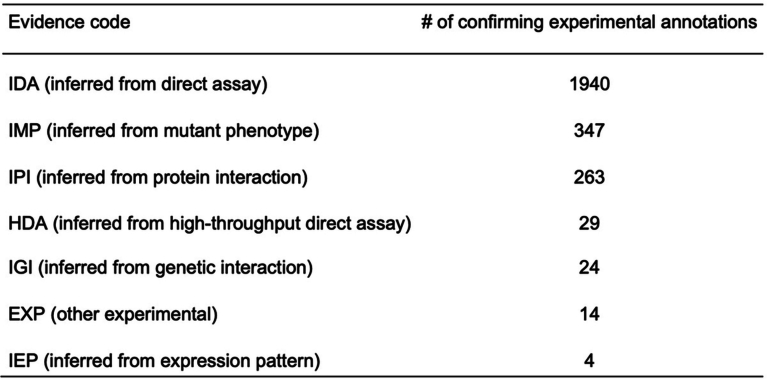
Type of experimental evidence for later GO annotations that confirmed earlier ‘predicted’ PAN-GO annotations

These are primary annotations added to the GO knowledgebase between October 2019 and March 2022, which were found to confirm (by direct experimental evidence) PAN-GO annotations made before October 2019 that were previously supported only by experimental evidence for a homologous gene. Detailed documentation of GO evidence codes can be found at https://geneontology.org/docs/guide-go-evidence-codes/.

## Data Availability

PAN-GO browser: https://functionome.geneontology.org. PAN-GO annotations: https://functionome.geneontology.org/download/functionome_release.gaf.gz. Evolutionary models: https://pantree.org, https://functionome.geneontology.org/download/IBD.gaf. Phylogenetic trees: https://data.pantherdb.org/ftp/panther_library/15.0/.

## Code availability

The following code and software are available from GitHub: website code, https://github.com/pantherdb/pango; and PAINT software (for constructing evolutionary models), https://github.com/pantherdb/db-PAINT.
