# Supplementary material for: A compendium of human gene functions derived from evolutionary modelling
Source: Nature. 2025 Feb 26;640(8057):146–54. doi: 10.1038/s41586-025-08592-0 (PMC11964926; doi:10.1038/s41586-025-08592-0)
Supplement: Supplementary file 1 — This file (PDF format) contains comparisons between PAN-GO human function annotations and GO annotations from other sources, including comparisons of GO enrichment analysis results. It comprises Supplementary Results, Supplementary Fig. 1 and Supplementary Tables 1–8. [file 41586_2025_8592_MOESM1_ESM.pdf]

nature portfolio

https://doi.org/10.1038/s41586-025-08592-0

Supplementary information

A compendium of human gene functions derived from evolutionary modelling

In the format provided by the authors and unedited

Supplementary Results

Comparing PAN-GO annotations to other sources of GO annotations

Gene Ontology (GO) annotations for human genes can be obtained both from the GO knowledgebase, and automatic function prediction (AFP) algorithms. These other sources differ from the PAN-GO set here, in that they have not been designed to represent the known repertoire of human gene functions as completely, concisely and accurately as possible. Predicted annotations in the GO knowledgebase, as well as from AFP methods, are not explicitly traceable to the experimental evidence supporting them. We have performed analyses to demonstrate how PAN-GO annotations differ from these other sources, and how they impact the widely used genomic technique of GO enrichment analysis.

Comparing to other annotations available in the GO knowledgebase

To characterize how the work reported here differs from the prior work of the GO Consortium, we performed a number of analyses to compare the PAN-GO annotation set to the other currently available GO annotation sets. These analyses demonstrate the value of the PAN-GO set in filling gaps in the previous sets of annotations, as well as excluding less informative annotations that are present in those previous sets. We show on previously published case studies that the PAN-GO annotation set removes or reduces the major bias that has been demonstrated to confound gene set enrichment analysis[1].

Coverage of annotations

The PAN-GO annotations cover more protein-coding genes (17,027) than do experimental annotations (EXP; 13,844), and slightly less than computationally predicted annotations (IEA; 17,804) (Extended Data Figure 2). We note that, while PAN-GO annotations must be traceable to experimental evidence, the same is not true of computationally predicted GO annotations. We examined the breadth of the annotation coverage of each human gene by counting the number of different GO aspects to which the gene is annotated (MF, BP and CC). The PAN-GO set has a larger number of genes that are covered by all three GO aspects, and by at least two aspects, than either the experimental or computationally predicted annotation sets.

Distribution of annotations across different genes

The distribution of annotations in the PAN-GO set is qualitatively and quantitatively different from the other sets. Qualitatively, it is a peaked distribution rather than monotonically decreasing, and quantitatively, it has a much shorter tail of genes with a large number of distinct GO terms (Extended Data Figure 3). Both of these properties show that PAN-GO annotations exhibit a much greater evenness of the annotation coverage across the genome, with fewer genes that have very few, or very many, distinct annotated functional characteristics. The peak in the PAN-GO set shows that a representative (mode) gene has 3-4 distinct GO terms describing its function, rather than 1 as for experimental or predicted annotations. Together with Extended Data Figure 2, this illustrates the extensive amount of additional information present in the PAN-GO annotations, compared to the GO annotations for human genes that were previously available. The shorter tail for PAN-GO in Extended Data Figure 3 shows that there are relatively few highly annotated genes (genes that are annotated to a large number of distinct GO terms) in the PAN-GO set, compared to experimental and computational annotations. In the PAN-GO annotation set, the top 10% most highly annotated genes account for 15% of the total annotations, while in the EXP set, the top 10% most highly annotated genes account for 37% of the total annotations. This bias in the experimental annotations is largely due to the bias in the experimental literature, rather than actual differences in the functional complexity of different genes. PAN-GO therefore dramatically reduces the number of annotations for genes that are highly annotated in other sets, which we explore further below.

GO terms used in PAN-GO compared to previously available GO annotations

Supplementary Figure 1 shows how the PAN-GO annotations compare to two types of previously available GO annotations. The first set is the experimental annotations (EXP) for human genes, which can be traced to experimental evidence, but, as described in the main text, generally each describe the conclusions from a single experiment. The second set is computationally predicted annotations (inferred from electronic annotation, IEA) for human genes, which are determined using a variety of methods including InterPro2GO[2] and via 1:1 orthology relations determined by Ensembl Compara[3]. Details and references for each of the prediction methods is given in Supplementary Table 1.

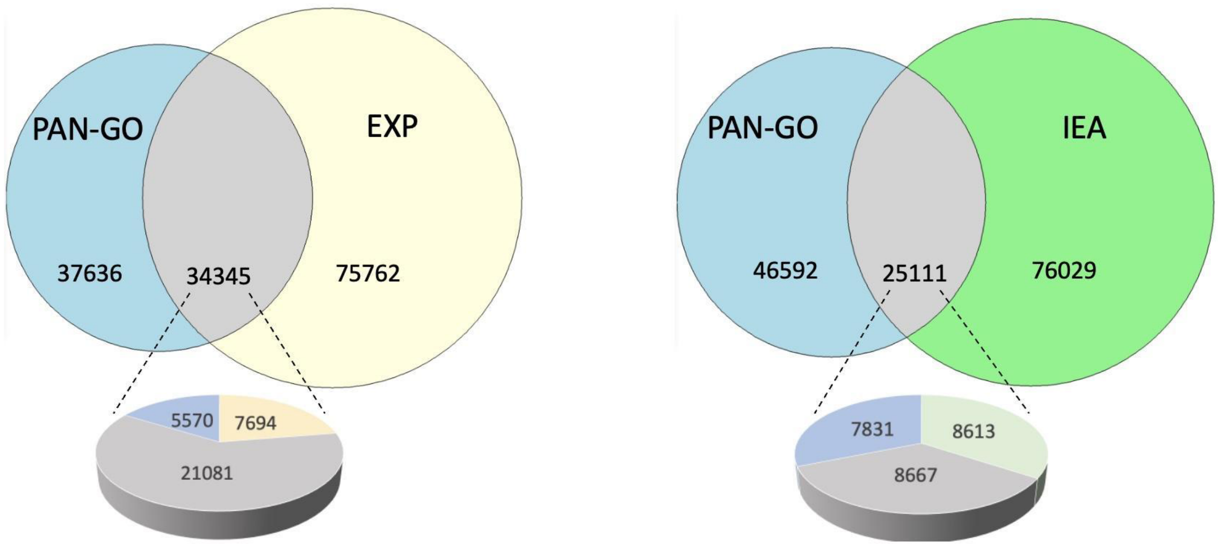

Supplementary Figure 1. Comparison of PAN-GO annotations to experimental (EXP) and predicted (IEA) GO annotations for human genes. The Venn diagrams show the number of annotations that are unique to each set, and the annotations that overlap to some degree between the sets (gray). In the case of overlap (gray), the pie charts show three different types of overlap: 1) identical (gray), i.e. the PAN-GO annotation is to the same GO term as in the other set, 2) PAN-GO more specific (blue), i.e. the PAN-GO annotation is to a related GO term that is more specific than in the other set, and 3) PAN-GO less specific, i.e. the PAN-GO annotation is to a related term that is less specific than in the other set.

Supplementary Table 1. GO term prediction (IEA) methods included in comparison. These are the methods currently used to produce predicted annotations included the GO knowledgebase. GO internal references (starting with GO_REF:) describe specific annotation methods and are available at https://github.com/geneontology/go-site/tree/master/metadata/gorefs/README.md.

Method	Reference	Brief description
Automatic transfer of experimentally verified manual GO annotation data to orthologs using Ensembl Compara	GO_REF:0000107	Homology, from experimental evidence propagated from one gene to one orthologous gene
Gene Ontology annotation through association of InterPro records with GO terms	GO_REF:0000002	Homology, from a hit to an InterPro signature
Gene Ontology annotation based on Enzyme Commission mapping	GO_REF:0000003	Imported from another resource, from mapping an EC number assigned in UniProt
Gene Ontology annotation based on UniProtKB keyword mapping	GO_REF:0000004	Imported from another resource, from mapping a manually assigned Swiss-Prot keyword
Electronic Gene Ontology annotations created by transferring manual GO annotations between related proteins based on shared sequence features	GO_REF:0000104	Homology, from manually curated UniRule
Automatic assignment of GO terms using logical inference, based on on inter-ontology links	GO_REF:0000108	Logical assertion using the ontology, from asserted relation between different aspects of GO
Electronic Gene Ontology annotations created by ARBA machine learning models	GO_REF:0000117	Computational, from machine learning

As shown in Supplementary Figure 1, over half (37,636) of the PAN-GO annotations are completely distinct from the experimental GO annotations (EXP); in addition, there are 5,570 PAN-GO annotations that are to a related term, but greater specificity, than any experimental annotation. In both of these cases, PAN-GO annotations add information beyond what was previously available. Interestingly, PAN-GO annotations overlap even less with predicted GO annotations (IEA) than with experimental GO annotations.

To better understand the differences between these sets of GO annotations, we identified the GO terms that appeared more commonly in the PAN-GO set compared to the EXP set (Supplementary Table 2), and vice versa (Supplementary Table 3). We first consider the terms that often appear in PAN-GO annotations but not EXP annotations for the same gene (Supplementary Table 2). For molecular function, three of the GO terms that appear most commonly in PAN-GO annotations but not EXP annotations for a given gene are related to the function, and type of regulatory region bound by, DNA binding transcription factors. This reflects the fact that many human transcription factors lack experimental evidence for their functions, and are supported only by experimental evidence for homologous genes. A similar situation obtains for olfactory receptors, which mostly remain experimentally uncharacterized. Three of the biological process terms frequently added by PAN-GO reflect the larger processes (transcription regulation and signaling) that correlate with the molecular functions above. In addition, the many genes involved in cell differentiation and the innate immune response are often only annotated in the PAN-GO set. In general, then, the PAN-GO set includes many important functions of human genes that have been highly studied in related genes in other organisms, but not directly for a human gene itself.

Supplementary Table 2. Most frequent GO terms in PAN-GO but not EXP for same gene

MF term	Number MF	BP term	Number BP	CC term	Number CC
GO:0000981 (DNA-binding transcription factor activity, RNA polymerase II-specific)	788	GO:0006357 (regulation of transcription by RNA polymerase II)	942	GO:0005634 (nucleus)	1170
GO:0000978 (RNA polymerase II cis-regulatory region sequence-specific DNA binding)	493	GO:0030154 (cell differentiation)	189	GO:0005737 (cytoplasm)	1000
GO:0004984 (olfactory receptor activity)	216	GO:0007186 (G protein-coupled receptor signaling pathway)	183	GO:0005886 (plasma membrane)	763
GO:0005509 (calcium ion binding)	152	GO:0007165 (signal transduction)	179	GO:0005615 (extracellular space)	568

GO:0000977 (RNA polymerase II transcription regulatory region sequence-specific DNA binding)	141	GO:0045087 (innate immune response)	172	GO:0005887 (integral component of plasma membrane)	409

We next consider the GO terms that often appear in EXP annotations but not PAN-GO annotations for the same gene (Supplementary Table 3). For molecular function, these are all subtypes of noncovalent binding functions. Two of these terms indicate that a protein binds to itself. While this is an important property of the functional structure of a protein (its "quaternary structure," e.g. dimer, trimer, etc.), it does not in itself represent an important functional characteristic, and is generally not used in PAN-GO annotations. The other terms often missing from the PAN-GO set (RNA binding, enzyme binding, protein kinase binding) are often parts of other functions that are in the PAN-GO set, so they would not represent independent functional characteristics even though they are not related in the GO ontology. For example, a *protein kinase inhibitor* may bind to a protein kinase (GO term: *protein kinase binding*) and alter the conformation of that kinase such that it does not function. If *protein kinase inhibitor* is selected for modeling, then *protein kinase binding* is considered to be a redundant characteristic. For biological process, the top terms with experimental evidence that are missing from the PAN-GO set are all regulation terms: positive and negative regulation of transcription, and positive regulation of cell proliferation. Primary annotations to these GO terms are often assigned based on experiments that knock out a gene or overexpress a gene, and measure downstream effects such as changes in the expression of other genes, or on cell growth and division. These effects are often "phenotypes" that can be far downstream, and very indirectly related to, the actual function of a gene. Because the PAN-GO process involves a review of all primary GO annotations in a family, PAN-GO curators will not select these terms for evolutionary modeling if, when considered together with all the function evidence in the family, those terms are judged to be indirect effects that arise from the annotated "core" functions of the gene. For GO cellular component, *extracellular exosome* is generally lacking from the PAN-GO set. This is because an exosome is very rarely the location in which a given gene product has been demonstrated to function, even if an experiment has shown it to be located there under certain conditions. In general, many primary annotations to GO cellular component terms are based on an experimental observation of subcellular localization under a particular condition; however, PAN-GO annotations strive to represent where the protein is functionally active, in accordance with the original specification of that aspect of the GO[4] and the definition of gene function[5]. The PAN-GO process of

considering all functional characteristics plays an important role in helping to select which location(s) are likely to be the ones in which a protein is active.

Supplementary Table 3. Most frequent GO terms in EXP but not PAN-GO for same gene

MF term	Number MF	BP term	Number BP	CC term	Number CC
GO:0042802 (identical protein binding)	1379	GO:0045893 (positive regulation of transcription, DNA-templated)	334	GO:0005654 (nucleoplasm)	2355
GO:0003723 (RNA binding)	797	GO:0045892 (negative regulation of transcription, DNA-templated)	256	GO:0070062 (extracellular exosome)	1959
GO:0042803 (protein homodimerization activity)	543	GO:0045944 (positive regulation of transcription by RNA polymerase II)	253	GO:0005829 (cytosol)	1940
GO:0019899 (enzyme binding)	305	GO:0010628 (positive regulation of gene expression)	245	GO:0005634 (nucleus)	1510
GO:0019901 (protein kinase binding)	262	GO:0008284 (positive regulation of cell population proliferation)	220	GO:0005737 (cytoplasm)	1347

We also identified the terms that most commonly appear in PAN-GO annotations compared to predicted (IEA) GO annotations for the same gene, and vice-versa. Supplementary Table 4 shows that PAN-GO tends to annotate transcription factor-related terms that are missing from the IEA annotations, as well as the molecular functions of *olfactory receptor* and *ubiquitin-protein ligase* activities, and the biological process of *innate immune response*. The IEA molecular function annotations (Supplemental Table 5) tend to favor terms that capture cofactor binding (*zinc*, and the generic *metal ion*) as well as *ATP binding*, which are considered in PAN-GO to be required for the function, but not actually representing the function itself. Many of the highly used IEA terms are very non-specific, and PAN-GO often contains much more specific terms instead; examples of such non-specific terms include *DNA binding*, *apoptotic process*, and *regulation of catalytic activity*. Interestingly, many of the same terms appear in both lists, indicating that they are used in both PAN-GO and predicted annotations, but there is disagreement about which genes are annotated with those terms. In many cases, these discrepancies can be explained by the fact that all PAN-GO annotations are traceable to specific experimental evidence, which is not the case for the IEA annotations.

Supplementary Table 4. Most frequent GO terms in PAN-GO but not IEA for same gene

MF term	Number MF	BP term	Number BP	CC term	Number CC
GO:0000978 (RNA polymerase II cis-regulatory region sequence-specific DNA binding)	830	GO:0006357 (regulation of transcription by RNA polymerase II)	681	GO:0005634 (nucleus)	2064
GO:0000981 (DNA-binding transcription factor activity, RNA polymerase II-specific)	825	GO:0045087 (innate immune response)	202	GO:0005737 (cytoplasm)	1507
GO:0000977 (RNA polymerase II transcription regulatory region sequence-specific DNA binding)	200	GO:0000122 (negative regulation of transcription by RNA polymerase II)	164	GO:0005886 (plasma membrane)	1008
GO:0004984 (olfactory receptor activity)	187	GO:0007165 (signal transduction)	153	GO:0005615 (extracellular space)	803
GO:0061630 (ubiquitin protein ligase activity)	169	GO:0007186 (G protein-coupled receptor signaling pathway)	142	GO:0005829 (cytosol)	704

Supplementary Table 5. Most frequent GO terms in IEA but not PAN-GO for same gene

MF term	Number MF	BP term	Number BP	CC term	Number CC
GO:0046872 (metal ion binding)	2480	GO:0007186 (G protein-coupled receptor signaling pathway)	518	GO:0016021 (integral component of membrane)	3639
GO:0005524 (ATP binding)	1356	GO:0050790 (regulation of catalytic activity)	406	GO:0005737 (cytoplasm)	1108
GO:0008270 (zinc ion binding)	539	GO:0006915 (apoptotic process)	384	GO:0005634 (nucleus)	1041
GO:0004930 (G protein-coupled receptor activity)	490	GO:0030154 (cell differentiation)	378	GO:0005886 (plasma membrane)	896
GO:0003677 (DNA binding)	467	GO:0050911 (detection of chemical stimulus involved in sensory perception of smell)	362	GO:0005576 (extracellular region)	664

Comparison of gene set enrichment analysis results

The most common use case of GO annotations is in gene set enrichment. Yet a major confounder of enrichment analysis was identified and extensively documented in a seminal study by Ballouz *et al.*[1], namely the presence of genes that are annotated to a large number of distinct GO terms. Highly annotated genes are often referred to as "multifunctional genes" and in some cases this is true, but as described above, in many cases primary annotations represent partial functions or downstream phenotypes rather than distinct functions. In their study, Ballouz *et al.* developed a method of

correcting for highly annotated genes, by retaining only the enriched GO terms that are robust to removing the most highly annotated genes.

There are no "gold standard" test sets for enrichment analysis. We therefore assessed the impact of using the PAN-GO annotations for GO enrichment analysis of three human gene sets that had been previously shown by Ballouz *et al*. to be biased by highly annotated genes, and used as detailed case studies. To perform our assessment, we added the PAN-GO annotation set to the PANTHER gene list analysis tool[6], and analyzed each gene list using either the PAN-GO annotations alone, or all GO annotations (including experimental annotations, computational annotations, and PAN-GO). We describe the results for each distinct gene set below, and compare our results to the analyses by Ballouz *et al*. for these same case studies. The enrichment analysis tool we used, which includes PAN-GO annotations, is available at functionome.geneontology.org.

Case study 1: Genomic copy number variants (CNVs) in autism

Gilman *et al*.[7] used GO enrichment analysis to advance the hypothesis that perturbations of synaptic development and function underlie the autistic phenotype. Analyzing the list of 72 genes with CNVs found in cases compared to controls, they found 16 GO biological process terms (at a false discovery rate threshold FDR<0.01), only a few of which were brain-related, including *learning and memory* and *neuron development*. To support the hypothesis of the involvement of synapses, these results were combined with GO cellular component term enrichment, which included *synapse*. When we re-analyzed their list of 72 genes using all GO annotations, we find significant enrichment (FDR<0.05) for 47 groups of biological process terms (each group represents GO terms that are related in the ontology, and therefore have many genes in common), including a group of terms including *learning and memory* as observed in the original paper and by Ballouz *et al*. Yet Ballouz *et al*. find that the *learning and memory* enrichment is a likely artifact of highly annotated genes, and they show that their correction for highly annotated genes removes this enrichment, as well as many other terms. Interestingly, when we re-analyzed the same list of all 72 genes (i.e. not removing highly annotated genes) using only PAN-GO annotations, *learning and memory* is no longer enriched, and we find only 6 groups of enriched biological process terms, 3 of which are synapse-related: *receptor clustering*, *postsynapse organization*, and *chemical synaptic transmission*. Thus, using PAN-GO in the gene set enrichment analysis excludes some of the same terms that were excluded by using Ballouz *et al*.'s multifunctionality correction, while identifying terms that support the conclusions drawn by the

authors of the original paper, but without requiring sifting through tens of other enriched term clusters that were not considered as biologically relevant by the authors.

Case study 2: Gene expression changes in response to hypoxia

Manalo *et al*.[8] identified genes which were both 1) induced in response to hypoxia, and 2) induced by a constitutively active form of the HIF-1 transcription factor. Their own analysis of the 202 genes in this list identified a preponderance of transcription factors, collagens, and genes involved in signal transduction. Ballouz *et al*. found that enrichment analysis using GO biological process annotations identifies a large number of enriched terms. However, they find that after removing highly annotated genes, a much smaller number of enriched processes remain statistically significant, including *peptidyl-proline modification*, *cellular response to hypoxia*, and *collagen fibril organization*, which, as they point out, capture the main conclusions of the authors of the original paper. Our own re-analysis found similar results. When reanalyzing with all GO biological process annotations, we found 85 groups of significantly enriched (FDR<0.05) terms. This large number makes interpretation difficult. When confining the analysis to using only the PAN-GO annotations, we found only 8 significantly enriched groups, including terms identical to, or related to, all of the above terms highlighted by Ballouz *et al*., as well as terms around transcriptional regulation which reflect the preponderance of transcription factors reported by Manalo *et al*. Again, enrichment analysis using the PAN-GO set is able to identify the main biologically relevant GO terms, while excluding many of the same terms that were excluded by the corrections proposed by Ballouz *et al*., yet without requiring any correction to be made.

Case study 3: Genome-wide association studies of schizophrenia

Schmidt-Kastner *et al*.[9] performed a meta-analysis of the published literature to identify 42 schizophrenia candidate genes. The authors performed enrichment analysis of this gene list and found many enriched terms, but the two with the smallest P-values were *synaptic transmission* and *developmental process*, and these were reported in the paper. However, after correcting for highly annotated genes, Ballouz *et al*. found that no biological process terms were enriched to a level that reached statistical significance, though *synaptic transmission* was nearly significant. Our re-analysis of these 42 genes using all GO annotations resulted in significant (FDR<0.05) enrichment for an astounding 140 distinct groups of biological process terms, covering a broad range of very different processes. Consistent with the initially published analysis, in our re-analysis the two terms

with the smallest P-values were *synaptic transmission*, and *nervous system development* (a more specific term than the initially reported *developmental process*). When we analyzed the same gene list using only PAN-GO annotations, we found 6 groups of biological processes to be significantly enriched (FDR<0.05), including 3 groups around even more specific and informative GO terms: *anterograde trans-synaptic transmission*, *modulation of trans-synaptic signaling*, and *neuron differentiation*. In this case, the enrichment results using PAN-GO are not only targeted toward the biological interpretation reported by the authors, but also they yield more specific and informative insights.

These case studies demonstrate that the PAN-GO annotations are valuable not only for what they add to the experimental annotations, but also for what they subtract, or exclude, from the set of GO annotations for each human gene.

Comparing PAN-GO annotations to automatic function prediction methods not in the GO knowledgebase

A number of automatic function prediction (AFP) methods have been developed, and many of these have been assessed in the Critical Assessment of Function Annotation (CAFA) competitions[10, 11]. The CAFA competitions have established assessment metrics for comparing the predictions from AFP methods. The basic procedure is to set a date cutoff for predictions (thus limiting the predictions to using only experimental GO annotations that were available before the date cutoff), and then use new experimental GO annotations that accrue after the cutoff date as a “test set” for assessment. Proxy measures of precision and recall are then calculated using the “protein-centric precision” and “protein-centric recall” as defined by Clark and Radivojac[12]. Because AFP methods include a prediction score (reflecting the relative certainty of the prediction) protein-centric precision and protein-centric recall can be calculated at different score thresholds, and F_{max}, the maximum F score (the harmonic mean of protein-centric precision and recall) across a range of score thresholds, is compared between AFP methods.

Although PAN-GO represents a curated integration and careful selection of GO terms in the context of phylogenetic models, and is not an AFP method, the PAN-GO annotations based only on

experimental evidence in homologous genes can be considered to be predictions, as they have not yet been established by direct experimental evidence. To compare these predictions in PAN-GO to AFP methods, we used the datasets we created for PAN-GO validation, as described in Methods. These datasets comprise a "test set" of annotations to be predicted (all newly accumulated experimental GO annotations after October 2019), a "training set" of experimental GO annotations (all experimental GO annotations prior to October 2019) and the set of PAN-GO predicted annotations as of October 2019 for evaluating against the test set. We then calculated protein-centric precision and protein-centric recall for PAN-GO (as of October 2019), as well as for different AFP methods. We selected several top-performing AFP methods from the CAFA assessment and from CAFA-like assessments reported in the subsequent literature.

Only one of these AFP methods, DeepGOZero[13], has made predicted annotations for human genes available for download, which we obtained at https://deepgo.cbrc.kaust.edu.sa/data/deepgozero/zero_predictions.tar.gz. For another method, PANNZER[14, 15], a web server is available at http://ekhidna2.biocenter.helsinki.fi/sanspanz/ that can accept all human protein coding gene sequences as input. For the other methods, to maximize comparability with PAN-GO from October 2019, for four additional AFP methods, DeepGOPlus[16], DeepGOCNN, DiamondScore, and TALE[17], we were able to install the code locally and retrain them using only the experimental GO annotations available prior to October 2019. The source code for DeepGOPlus, including the DeepGOCNN and DiamondScore modules, was downloaded from https://github.com/bio-ontology-research-group/deepgoplus. TALE was downloaded from https://github.com/Shen-Lab/TALE. Protein-centric precision, recall and F scores for each method are shown in Supplementary Table 6 (MF), Supplementary Table 7 (CC) and Supplementary Table 8 (BP); code for the calculations is provided at https://github.com/geneontology/PAN-GO_CAFA_evaluation. For PAN-GO, there is only a single F score, but AFP methods include confidence scores and Supplementary Tables 6-8 show the results for all deciles of the confidence score.

As shown in Supplementary Tables 6, 7 and 8, using the F_{max} metric from Clark and Radivojac, the single PAN-GO F score is greater than the F_{max} for all AFP methods. This holds for all three aspects of GO, molecular function (MF, Supplementary Table 6), cellular component (CC, Supplementary Table 7) and biological process (BP, Supplementary Table 8). After PAN-GO, the more recently

developed methods (DeepGOPlus, DeepGOCNN, DiamondScore and TALE) have the next best F_{max} values, despite the fact that the PANNZER web server was updated in June 2024 and in principle has access to the experimental annotations in the test set. Although it is also a more recent method, DeepGOZero performs poorly on our test set, which is expected because these are “zero-shot” predictions designed to predict GO terms that are rarely annotated in the GO knowledgebase.

We found the superior performance of PAN-GO surprising, as there are several reasons to expect that a CAFA-like evaluation would tend to underestimate the performance of PAN-GO. First, PAN-GO does not include confidence scores so only a single F score is available, while for AFP methods information in the test set is used to select the best F score among a range of score thresholds. But even more importantly, in the CAFA-like evaluation process, experimental annotations (accrued during a post-prediction time period) are treated as the absolute “true” annotations to be predicted. As described in the main text and Methods, PAN-GO annotations are selective, meaning that many experimental GO annotations for human genes (and related genes) have been intentionally left out of the PAN-GO set, when they are deemed to reflect redundant functional characteristics even if they appear in distinct branches of the ontology. Consequently, PAN-GO predictions should manifest an artificially low true positive rate (and therefore precision, recall and F score) on any test set composed of accrued experimental annotations. We interpret the surprisingly good performance of PAN-GO despite these drawbacks with caution, as the analysis focuses on only a single metric, for a single test set. Nevertheless, we note that PAN-GO is carefully curated and is designed to produce a select set of highly accurate, minimally redundant GO annotations. It has already been suggested that, rather than comparing to AFP methods, PAN-GO could instead be used to help assess AFP methods, and a previous study[18] demonstrated that a carefully constructed test set that balances both positive and negative annotations from PAN-GO can be used to estimate actual false positive rates, a known issue with the current CAFA metrics[19].

Supplementary Tables 6, 7 and 8 also (right hand columns) show a comparison between the GO annotations from PAN-GO, and the predicted annotations from each AFP method at different score thresholds. Predicted annotations are first made non-redundant as described in Extended Data Figure 3. As expected, the degree of overlap with PAN-GO depends on the score threshold, but in all cases there are many PAN-GO annotations that are not predicted by a given AFP method. Among the AFP methods, PANNZER shows the greatest annotation overlap with PAN-GO at all score thresholds.

Except at the lowest score thresholds for some AFP methods, PAN-GO tends to produce a much smaller number of annotations than any AFP method, in keeping with its property of being highly selective, including only the most informative annotations.

Supplementary Table 6. Comparison of molecular function (MF) annotations from PAN-GO to those generated by automatic prediction methods.

For AFP methods, confidence scores are available so all values are calculated for different threshold fractions t of all predicted annotations. Precision, recall and F score are calculated using the definitions of Clark and Radivojac, treating experimental annotations for human genes accrued after the prediction date as the test set, as described above and in Methods. Bold indicates F_{max}, the maximum F score (balancing precision and recall) for each annotation set. The comparison of annotated GO terms is shown in the rightmost columns. GO terms may be identical, have ancestor-descendant relationships in the ontology (one may be more specific than the other), or they may be unique to one method or the other.

		Performance on MF test set			MF annotation comparison with PAN-GO				
Method	t	Protein-centric Precision	Protein-centric Recall	Protein-centric F score	Identical annotations	PAN-GO more specific	AFP method more specific	Unique to PAN-GO	Unique to AFP method
PAN-GO	-	0.497	0.512	**0.504**					
DeepGOPlus	0.1	0.525	0.104	0.174	298	1438	57	7152	1036
	0.2	0.487	0.182	0.265	772	1880	199	6096	1989
	0.3	0.455	0.252	0.324	1308	1962	353	5335	3024
	0.4	0.426	0.315	0.362	1859	1995	492	4622	4214
	0.5	0.397	0.363	0.379	2358	1928	653	4049	5630
	0.6	0.377	0.400	0.388	2882	1837	809	3494	7215
	0.7	0.364	0.433	**0.396**	3443	1685	962	2967	8977
	0.8	0.344	0.464	0.395	3830	1561	1090	2598	11064
	0.9	0.327	0.493	0.393	4147	1429	1235	2310	13105
	1	0.309	0.513	0.386	4443	1329	1365	2016	15225
DeepGOCNN	0.1	0.443	0.095	0.157	183	1174	62	7396	1147
	0.2	0.425	0.146	0.218	336	1916	150	6417	2649
	0.3	0.392	0.185	0.252	474	2333	215	5803	4279
	0.4	0.371	0.223	0.279	637	2692	293	5215	5799
	0.5	0.352	0.266	0.303	805	2981	393	4667	7319
	0.6	0.342	0.303	0.322	972	3175	502	4225	8809
	0.7	0.331	0.341	0.336	1141	3365	604	3775	10173
	0.8	0.324	0.382	0.351	1378	3522	703	3306	11563
	0.9	0.317	0.416	0.360	1717	3525	807	2882	12764
	1	0.315	0.448	**0.370**	2117	3365	953	2514	13731

DiamondScore	0.1	0.521	0.077	0.135	415	744	113	7504	762
	0.2	0.486	0.168	0.250	1006	1041	234	6502	1623
	0.3	0.459	0.245	0.319	1560	1249	349	5633	2506
	0.4	0.430	0.307	0.358	2162	1244	494	4903	3516
	0.5	0.401	0.351	0.374	2834	1137	675	4196	4594
	0.6	0.382	0.387	0.384	3409	1059	849	3547	6020
	0.7	0.362	0.427	0.392	3872	922	993	3103	7688
	0.8	0.349	0.458	0.396	4225	856	1132	2704	9319
	0.9	0.339	0.494	**0.402**	4389	1048	1242	2257	11287
	1	0.324	0.524	0.400	4436	1302	1334	1891	13719
DeepGOZero	0.1	0	0	na	0	0	1	2697	309
	0.2	0	0	na	0	0	27	2674	620
	0.3	0	0	na	0	0	48	2659	957
	0.4	0	0	na	0	0	59	2648	1253
	0.5	0	0	na	0	0	68	2641	1534
	0.6	0	0	na	0	0	79	2635	1853
	0.7	0	0	na	0	0	82	2634	2145
	0.8	0	0	na	0	0	89	2627	2458
	0.9	0	0	na	0	0	91	2625	2757
	1	0	0	na	0	0	94	2624	2986
PANNZER	0.1	0.268	0.038	0.067	693	104	151	8499	1577
	0.2	0.273	0.079	0.122	1423	190	300	7547	3128
	0.3	0.267	0.114	0.160	2126	270	449	6654	4689
	0.4	0.265	0.149	0.191	2822	334	608	5772	6249
	0.5	0.273	0.185	0.220	3487	407	783	4912	7749
	0.6	0.277	0.223	0.247	4165	479	927	4067	9273
	0.7	0.279	0.257	0.268	4870	547	1089	3200	10817
	0.8	0.283	0.290	0.286	5551	601	1246	2388	12396
	0.9	0.290	0.330	0.309	6206	655	1390	1604	13912
	1	0.291	0.362	**0.322**	6894	701	1558	800	15475
TALE	0.1	0.318	0.535	**0.399**	1785	1496	1264	4206	19772
	0.2	0.201	0.618	0.303	2284	1479	1983	3413	44066
	0.3	0.153	0.678	0.250	2620	1329	2549	3026	67918
	0.4	0.124	0.718	0.211	2859	1245	3060	2726	91674
	0.5	0.105	0.739	0.183	3049	1186	3554	2462	115690
	0.6	0.090	0.759	0.160	3217	1101	4059	2264	139712
	0.7	0.078	0.771	0.141	3350	1017	4583	2107	163612
	0.8	0.067	0.783	0.123	3456	913	5120	2011	187772
	0.9	0.057	0.790	0.106	3513	852	5835	1930	212576
	1	0.046	0.797	0.088	3529	801	7113	1852	240918

Supplementary Table 7. Comparison of cellular component (CC) annotations from PAN-GO to those generated by automatic prediction methods.

For AFP methods, confidence scores are available so all values are calculated for different threshold fractions t of all predicted annotations. Precision, recall and F score are calculated using the definitions of Clark and Radivojac, treating experimental annotations for human genes accrued after the prediction date as the test set, as described above and in Methods. Bold indicates F_{max}, the maximum F score (balancing precision and recall) for each annotation set. The comparison of annotated GO terms is shown in the rightmost columns. GO terms may be identical, have ancestor-descendant relationships in the ontology (one may be more specific than the other), or they may be unique to one method or the other.

		Performance on CC test set			CC annotation comparison with PAN-GO				
Method	t	Protein-centric Precision	Protein-centric Recall	Protein-centric F score	Identical annotations	PAN-GO more specific	AFP method more specific	Unique to PAN-GO	Unique to AFP method
PAN-GO	-	0.481	0.464	**0.473**	-	-	-	-	
DeepGOPlus	0.1	0.629	0.101	0.174	702	3457	108	5224	1444
	0.2	0.541	0.194	0.286	1468	3526	337	4183	3784
	0.3	0.456	0.264	0.334	1995	3258	696	3612	6662
	0.4	0.398	0.322	0.356	2332	2929	1186	3231	9673
	0.5	0.368	0.375	0.372	2743	2702	1624	2722	12781
	0.6	0.345	0.425	0.381	3056	2531	2109	2250	15931
	0.7	0.323	0.467	**0.382**	3245	2351	2644	1916	18982
	0.8	0.301	0.502	0.376	3435	2072	3227	1712	22147
	0.9	0.283	0.531	0.370	3528	1911	3813	1506	25422
	1	0.270	0.558	0.364	3665	1757	4406	1304	28736
DeepGOCNN	0.1	0.611	0.100	0.172	562	3713	83	5132	1570
	0.2	0.491	0.170	0.253	1112	3494	220	4679	4211
	0.3	0.408	0.223	0.288	1456	3199	450	4427	7061
	0.4	0.364	0.278	0.315	1658	3008	789	4129	10003
	0.5	0.326	0.324	0.325	1734	2892	1262	3814	12932
	0.6	0.309	0.367	0.335	1738	2946	1747	3446	16091
	0.7	0.289	0.404	0.337	1764	3109	2341	2930	19324
	0.8	0.280	0.444	0.343	1977	3249	2886	2295	22634
	0.9	0.276	0.486	0.352	2323	3172	3433	1735	25811
	1	0.272	0.529	**0.359**	2804	2675	4040	1448	28233
DiamondScore	0.1	0.553	0.081	0.141	644	1803	207	6694	1430
	0.2	0.516	0.167	0.253	1424	2291	536	5177	3052
	0.3	0.484	0.254	0.333	2196	2264	917	4102	4849
	0.4	0.433	0.313	0.363	2843	1941	1348	3433	7017
	0.5	0.403	0.363	0.382	3297	1710	1791	2911	9341
	0.6	0.372	0.403	0.387	3622	1491	2357	2464	11749

	0.7	0.347	0.437	0.387	3777	1450	2808	2090	14655
	0.8	0.332	0.479	**0.392**	3848	1487	3226	1760	17976
	0.9	0.313	0.511	0.388	3807	1576	3694	1483	21101
	1	0.294	0.541	0.381	3724	1710	4053	1272	24243
DeepGOZero	0.1	0	0	na	0	0	285	8658	1863
	0.2	0	0	na	0	0	670	8290	4255
	0.3	0	0	na	0	0	1019	7962	6719
	0.4	0	0	na	0	0	1189	7848	9211
	0.5	0	0	na	0	0	1316	7782	12074
	0.6	0	0	na	0	0	1701	7768	14961
	0.7	0	0	na	0	0	1752	7730	18058
	0.8	0	0	na	0	0	1929	7627	20360
	0.9	0	0	na	0	0	2479	7444	22938
	1	0	0	na	0	0	2750	7318	25053
PANNZER	0.1	0.255	0.032	0.057	667	154	141	8424	1684
	0.2	0.267	0.061	0.100	1305	286	282	7532	3414
	0.3	0.274	0.093	0.139	1942	415	410	6664	5183
	0.4	0.296	0.129	0.180	2652	554	543	5732	6877
	0.5	0.300	0.163	0.211	3344	672	677	4843	8567
	0.6	0.303	0.193	0.236	4002	799	825	3977	10291
	0.7	0.300	0.223	0.256	4636	894	944	3179	12073
	0.8	0.304	0.252	0.276	5318	1011	1076	2310	13763
	0.9	0.302	0.282	0.292	5962	1104	1210	1497.000	15455
	1	0.305	0.313	**0.309**	6644	1174	1350	690.000	17167
TALE	0.1	0.386	0.430	**0.407**	1701	2271	1811	2828	14359
	0.2	0.271	0.617	0.376	1780	1970	3999	1979	30500
	0.3	0.203	0.700	0.314	1825	1659	6179	1642	47717
	0.4	0.161	0.748	0.265	1870	1405	8443	1482	65431
	0.5	0.132	0.779	0.226	1877	1256	10709	1341	83282
	0.6	0.112	0.802	0.196	1885	1147	12966	1251	100931
	0.7	0.096	0.820	0.172	1877	1047	15220	1165	118175
	0.8	0.084	0.833	0.152	1865	960	17601	1108	135373
	0.9	0.073	0.843	0.134	1806	902	19899	1055	152181
	1	0.066	0.848	0.122	1781	839	22384	1042	168837

Supplementary Table 8. Comparison of biological process (BP) annotations from PAN-GO to those generated by automatic prediction methods.

For AFP methods, confidence scores are available so all values are calculated for different threshold fractions t of all predicted annotations. Precision, recall and F score are calculated using the definitions of Clark and Radivojac, treating experimental annotations for human genes accrued after the prediction date as the test set, as described above and in Methods. Bold indicates F_{max}, the maximum F score (balancing precision and recall) for each annotation set. The comparison of annotated GO terms is shown in the rightmost columns. GO terms may be identical, have ancestor-descendant relationships in the ontology (one may be more specific than the other), or they may be unique to one method or the other.

		Performance on BP test set			BP annotation comparison with PAN-GO				
Method	t	Protein-centric Precision	Protein-centric Recall	Protein-centric F score	Identical annotations	PAN-GO more specific	AFP method more specific	Unique to PAN-GO	Unique to AFP method
PAN-GO	-	0.334	0.303	**0.318**					
DeepGOPlus	0.1	0.311	0.095	0.146	592	5238	321	10313	10751
	0.2	0.303	0.179	0.225	1318	6943	837	7489	23751
	0.3	0.271	0.246	0.258	2038	7189	1355	6169	38536
	0.4	0.243	0.297	0.268	2771	6904	1886	5375	54111
	0.5	0.223	0.339	**0.269**	3382	6629	2419	4762	70540
	0.6	0.208	0.374	0.267	4087	6202	3010	4168	86269
	0.7	0.193	0.403	0.261	4660	5893	3625	3631	102452
	0.8	0.182	0.428	0.256	5126	5489	4207	3294	118641
	0.9	0.172	0.453	0.250	5526	5044	4875	3059	134676
	1	0.162	0.474	0.242	5815	4702	5546	2856	150837
DeepGOCNN	0.1	0.306	0.075	0.120	299	5096	293	10790	12850
	0.2	0.315	0.136	0.190	557	7265	632	8170	29296
	0.3	0.273	0.184	0.220	766	8040	942	7020	47399
	0.4	0.240	0.229	0.234	1014	8725	1285	5918	66093
	0.5	0.222	0.269	0.243	1232	9199	1742	5017	84915
	0.6	0.203	0.307	**0.244**	1523	9279	2198	4440	102568
	0.7	0.189	0.340	0.243	1815	9284	2713	3931	119445
	0.8	0.181	0.371	0.243	2140	9042	3243	3597	134240
	0.9	0.174	0.400	0.243	2544	8615	3822	3328	145312
	1	0.168	0.431	0.242	3326	7753	4405	3099	152423
DiamondScore	0.1	0.296	0.094	0.143	825	3638	418	11271	8175
	0.2	0.277	0.172	0.213	1742	4987	851	8653	18448
	0.3	0.254	0.240	0.247	2578	5188	1421	7176	30298
	0.4	0.233	0.290	0.258	3370	5069	1951	6152	42833
	0.5	0.211	0.331	**0.258**	4100	4599	2531	5551	55771
	0.6	0.193	0.360	0.251	4677	4235	3144	5034	68584

	0.7	0.182	0.392	0.249	5081	3785	3884	4694	81448
	0.8	0.171	0.414	0.242	5365	3669	4538	4249	96147
	0.9	0.161	0.441	0.236	5640	3915	5069	3621	117968
	1	0.158	0.471	0.237	5812	4450	5512	2813	143393
DeepGOZero	0.1	4.91E-06	7.07E-06	**5.79E-06**	0	0	149	16056	30727
	0.2	1.51E-06	7.07E-06	2.49E-06	0	0	249	16011	60596
	0.3	1.86E-06	1.41E-05	3.29E-06	0	0	385	15985	88453
	0.4	1.41E-06	1.41E-05	2.57E-06	0	0	501	15964	115977
	0.5	1.19E-06	1.41E-05	2.19E-06	0	0	591	15944	141425
	0.6	9.88E-07	1.41E-05	1.85E-06	0	0	719	15917	167681
	0.7	1.71E-06	2.97E-05	3.23E-06	0	0	842	15897	194520
	0.8	1.58E-06	2.97E-05	3.00E-06	0	0	980	15838	222637
	0.9	1.47E-06	2.97E-05	2.80E-06	0	0	1071	15828	250832
	1	1.38E-06	2.97E-05	2.63E-06	0	0	1176	15808	277859
PANNZER	0.1	0.191	0.017	0.032	1068	261	221	14621	5203
	0.2	0.202	0.036	0.061	2123	460	411	13216	10420
	0.3	0.210	0.049	0.080	3141	643	628	11852	15591
	0.4	0.215	0.065	0.100	4203	823	845	10460	20767
	0.5	0.223	0.079	0.117	5277	983	1030	9110	25994
	0.6	0.230	0.095	0.135	6337	1125	1239	7777	31123
	0.7	0.234	0.108	0.148	7359	1268	1438	6501	36315
	0.8	0.233	0.121	0.159	8394	1394	1645	5239	41562
	0.9	0.235	0.139	0.174	9433	1491	1845	4023	46666
	1	0.238	0.153	**0.186**	10528	1577	2052	2760	51804
TALE	0.1	0.455	0.127	0.198	258	8029	152	5924	21085
	0.2	0.348	0.229	0.276	425	7652	301	6039	43672
	0.3	0.292	0.307	**0.299**	543	7464	496	6010	65278
	0.4	0.254	0.355	0.296	675	7578	708	5680	86772
	0.5	0.225	0.391	0.286	778	7779	897	5296	108478
	0.6	0.203	0.422	0.274	839	7898	1063	5029	129917
	0.7	0.184	0.443	0.260	887	7999	1192	4812	150727
	0.8	0.168	0.463	0.246	941	8019	1297	4684	171574
	0.9	0.154	0.475	0.232	1029	7986	1390	4582	191359
	1	0.146	0.484	0.225	1105	7927	1540	4519	212387

References

(1) Ballouz, S.; Pavlidis, P.; Gillis, J. Using predictive specificity to determine when gene set analysis is biologically meaningful. Nucleic Acids Res 2017, 45 (4), e20. DOI: 10.1093/nar/gkw957.

(2) Burge, S.; Kelly, E.; Lonsdale, D.; Mutowo-Muellenet, P.; McAnulla, C.; Mitchell, A.; Sangrador-Vegas, A.; Yong, S. Y.; Mulder, N.; Hunter, S. Manual GO annotation of predictive protein signatures: the InterPro approach to GO curation. Database (Oxford) 2012, 2012, bar068. DOI: 10.1093/database/bar068.

(3) Boeckmann, B.; Robinson-Rechavi, M.; Xenarios, I.; Dessimoz, C. Conceptual framework and pilot study to benchmark phylogenomic databases based on reference gene trees. Brief Bioinform 2011, 12 (5), 423-435. DOI: 10.1093/bib/bbr034.

(4) Ashburner, M.; Ball, C. A.; Blake, J. A.; Botstein, D.; Butler, H.; Cherry, J. M.; Davis, A. P.; Dolinski, K.; Dwight, S. S.; Eppig, J. T.; et al. Gene ontology: tool for the unification of biology. The Gene Ontology Consortium. Nat Genet 2000, 25 (1), 25-29. DOI: 10.1038/75556.

(5) Thomas, P. D. The Gene Ontology and the Meaning of Biological Function. Methods Mol Biol 2017, 3743-3741_3742.

(6) Mi, H.; Muruganujan, A.; Huang, X.; Ebert, D.; Mills, C.; Guo, X.; Thomas, P. D. Protocol Update for large-scale genome and gene function analysis with the PANTHER classification system (v.14.0). Nat Protoc 2019, 14 (3), 703-721. DOI: 10.1038/s41596-019-0128-8.

(7) Gilman, S. R.; Iossifov, I.; Levy, D.; Ronemus, M.; Wigler, M.; Vitkup, D. Rare de novo variants associated with autism implicate a large functional network of genes involved in formation and function of synapses. Neuron 2011, 70 (5), 898-907. DOI: 10.1016/j.neuron.2011.05.021.

(8) Manalo, D. J.; Rowan, A.; Lavoie, T.; Natarajan, L.; Kelly, B. D.; Ye, S. Q.; Garcia, J. G.; Semenza, G. L. Transcriptional regulation of vascular endothelial cell responses to hypoxia by HIF-1. Blood 2005, 105 (2), 659-669. DOI: 10.1182/blood-2004-07-2958.

(9) Schmidt-Kastner, R.; van Os, J.; Esquivel, G.; Steinbusch, H. W.; Rutten, B. P. An environmental analysis of genes associated with schizophrenia: hypoxia and vascular factors as interacting elements in the neurodevelopmental model. Mol Psychiatry 2012, 17 (12), 1194-1205. DOI: 10.1038/mp.2011.183.

(10) Radivojac, P.; Clark, W. T.; Oron, T. R.; Schnoes, A. M.; Wittkop, T.; Sokolov, A.; Graim, K.; Funk, C.; Verspoor, K.; Ben-Hur, A.; et al. A large-scale evaluation of computational protein function prediction. Nat Methods 2013, 10 (3), 221-227. DOI: 10.1038/nmeth.2340.

(11) Zhou, N.; Jiang, Y.; Bergquist, T. R.; Lee, A. J.; Kacsoh, B. Z.; Crocker, A. W.; Lewis, K. A.; Georghiou, G.; Nguyen, H. N.; Hamid, M. N.; et al. The CAFA challenge reports improved protein function prediction and new functional annotations for hundreds of genes through experimental screens. Genome Biol 2019, 20 (1), 244. DOI: 10.1186/s13059-019-1835-8.

(12) Clark, W. T.; Radivojac, P. Information-theoretic evaluation of predicted ontological annotations. Bioinformatics 2013, 29 (13), i53-61. DOI: 10.1093/bioinformatics/btt228.

(13) Kulmanov, M.; Hoehndorf, R. DeepGOZero: improving protein function prediction from sequence and zero-shot learning based on ontology axioms. Bioinformatics 2022, 38 (Suppl 1), i238-i245. DOI: 10.1093/bioinformatics/btac256.

(14) Koskinen, P.; Törönen, P.; Nokso-Koivisto, J.; Holm, L. PANNZER: high-throughput functional annotation of uncharacterized proteins in an error-prone environment. Bioinformatics 2015, 31 (10), 1544-1552. DOI: 10.1093/bioinformatics/btu851.

(15) Törönen, P.; Holm, L. PANNZER-A practical tool for protein function prediction. Protein Sci 2022, 31 (1), 118-128. DOI: 10.1002/pro.4193.

(16) Kulmanov, M.; Hoehndorf, R. DeepGOPlus: improved protein function prediction from sequence. Bioinformatics 2020, 36 (2), 422-429. DOI: 10.1093/bioinformatics/btz595.

(17) Cao, Y.; Shen, Y. TALE: Transformer-based protein function Annotation with joint sequence-Label Embedding. Bioinformatics 2021, 37 (18), 2825-2833. DOI: 10.1093/bioinformatics/btab198.
(18) Warwick Vesztrocy, A.; Dessimoz, C. Benchmarking gene ontology function predictions using negative annotations. Bioinformatics 2020, 36 (Suppl_1), i210-i218. DOI: 10.1093/bioinformatics/btaa466.
(19) Dessimoz, C.; Škunca, N.; Thomas, P. D. CAFA and the open world of protein function predictions. Trends Genet 2013, 29 (11), 609-610. DOI: 10.1016/j.tig.2013.09.005.
